# Evaluating vaccination strategies for reducing infant respiratory syncytial virus infection in low-income settings

**DOI:** 10.1186/s12916-015-0283-x

**Published:** 2015-03-10

**Authors:** Piero Poletti, Stefano Merler, Marco Ajelli, Piero Manfredi, Patrick K Munywoki, D James Nokes, Alessia Melegaro

**Affiliations:** Dondena Centre for Research on Social Dynamics and Public Policy, Department of Policy Analysis and Public Management, Universitá Commerciale L. Bocconi, via Rontgen n. 1, Milan, Italy; Center for Information Technology, Bruno Kessler Foundation, via Sommarive, 18, Trento, Italy; Department of Statistics and Mathematics Applied to Economics, University of Pisa, via Ridolfi 10, Pisa, Italy; Kenyan Medical Research Institute (KEMRI) - Wellcome Trust Research Programme, Centre for Geographic Medicine Research (Coast), Hospital Road, Kilifi, Kenya; School of Life Sciences and WIDER, University of Warwick, Coventry CV4 7AL, Warwick, UK

**Keywords:** Computational models, Infectious diseases, RSV, Vaccination, Household transmission

## Abstract

**Background:**

Respiratory syncytial virus (RSV) is a leading cause of lower respiratory tract disease and related hospitalization of young children in least developed countries. Individuals are repeatedly infected, but it is the first exposure, often in early infancy, that results in the vast majority of severe RSV disease. Unfortunately, due to immunological immaturity, infants are a problematic RSV vaccine target. Several trials are ongoing to identify a suitable candidate vaccine and target group, but no immunization program is yet in place.

**Methods:**

In this work, an individual-based model that explicitly accounts for the socio-demographic population structure is developed to investigate RSV transmission patterns in a rural setting of Kenya and to evaluate the potential effectiveness of alternative population targets in reducing RSV infant infection.

**Results:**

We find that household transmission is responsible for 39% of infant infections and that school-age children are the main source of infection within the household, causing around 55% of cases. Moreover, assuming a vaccine-induced protection equivalent to that of natural infection, our results show that annual vaccination of students is the only alternative strategy to routine immunization of infants able to trigger a relevant and persistent reduction of infant infection (on average, of 35.6% versus 41.5% in 10 years of vaccination). Interestingly, if vaccination of pregnant women boosts maternal antibody protection in infants by an additional 4 months, RSV infant infection will be reduced by 31.5%.

**Conclusions:**

These preliminary evaluations support the efforts to develop vaccines and related strategies that go beyond targeting vaccines to those at highest risk of severe disease.

**Electronic supplementary material:**

The online version of this article (doi:10.1186/s12916-015-0283-x) contains supplementary material, which is available to authorized users.

## Background

Respiratory syncytial virus (RSV) is characterized worldwide by recurrent epidemics [1-4] and represents a leading cause of hospitalization of young children in least developed countries (LDCs) [5-7], where the vast majority of severe disease (and deaths) caused by RSV occurs [6]. Indeed, RSV can be considered one of the predominant viral pathogens among hospitalized infants [5,7].

Primary RSV infection usually arises in the first two years of life, and repeated reinfections occur throughout life [3,4,8,9]. However, most severe disease occurs among individuals infected during their first year of life, usually as a consequence of primary RSV infection [3-6,8,10-12].

During the 1960s, trials of a formalin-inactivated RSV vaccine administered to RSV-naive US children led to enhanced lower respiratory tract disease (LRTD) following natural RSV challenge in the immunized group [13]. After the failure of this immunization experience, different live attenuated RSV vaccines were evaluated in clinical trials in adults, young children, and infants, but none has gone beyond early-stage clinical trials or been licensed yet [14]. Indeed, although the highest priority target population for vaccine development is the RSV-naive child [3,14], clinical evaluation of various vaccine candidates has met a range of problems related to the lack of immunological maturity and vaccine tolerance in this vulnerable age group that has thwarted progress [13,14]. It is still unclear which vaccine would result in an effective protection against RSV infection and RSV-associated disease, and which, if any, would be suitable for being administered safely to infants.

The first objective of this work is to investigate, through mathematical modeling techniques, RSV transmission patterns in a low-income setting in order to realistically describe and understand the transmission chain leading to RSV infant infection, which causes most of RSV-associated serious disease. An empirical basis for this work is household studies [9,15] that have suggested a significant role of school-age children in introducing and spreading RSV infection in the household. The second objective of this work is to assess which immunization strategies can be effective in preventing RSV at a population level. More specifically, we aim to identify which subgroup of the population should be targeted, and with which schedule, in order to achieve an effective reduction of RSV infection rates in those age groups at highest risk of severe disease. The exploration of the various alternatives might provide useful indications for current and future vaccine development efforts by assessing which age targets can be considered as valuable for interrupting the chain of transmission and reducing infection rates in the first year of life.

## Methods

Individual-based transmission models of infectious diseases represent a powerful tool to evaluate and optimize interventions aimed at controlling human infectious diseases [16-19]. The need for detailed epidemiological and socio-demographic data to calibrate micro-simulation models makes the implementation of similar tools for LDCs a modeling challenge. In this work, a highly detailed socio-demographic and disease transmission model is developed and used to investigate the RSV transmission dynamics in a predominantly rural location of a low-income setting of coastal Kenya, representing an illustrative case of a high-burden region with limited financial resources. Specifically, the RSV infection process is simulated over a synthetic population of individuals structured in households and schools that is consistent with the main descriptive statistics of the Kenyan population as reported in the Demographic and Health Surveys (DHS, [20], accessed Jul 2014).

### Modeling socio-demographic characteristics of a sub-Saharan population

The simulated synthetic population consists of about 200,000 individuals, all explicitly represented in the model and grouped into households and primary schools. In particular, the simulated households structure mirrors existing generational age gaps between parents and their children and the observed complex heterogeneity in the Kenyan population, such as the co-location of several generations (up to four) within a single household. This was achieved through intensive use of available socio-demographic data for Kenya from the Demographic and Health Surveys (DHS) and other available data. DHS data were used to randomly assign age and co-locate individuals in households. Individuals were assigned to schools according to the age-specific school attendance rates computed by using the DHS dataset and school size distribution as provided by the Open Kenya dataset ([21], accessed Jun 2014). Annual fertility rates by age were computed according to the DHS statistics for the years 2002-2008. Annual mortality rates by age were obtained from the World Population Prospects of the United Nations ([22], accessed Jun 2014). The model was capable of reproducing the observed distributions of both household and school size, the age structure of the Kenyan population and the age-specific school attendance rates. Further details on the procedure used for generating the synthetic population and the validation process against the available socio-demographic data are provided in Additional file 1.

### RSV transmission model

The epidemiological model distinguishes between primary RSV infection, related to the first exposure to RSV in life, and subsequent infections. In agreement with epidemiological evidence [4] and with past modeling works [23-25], temporary (waning) immunity is combined in our model with a lifelong reduced susceptibility of individuals who have already experienced a first RSV infection.

The transmission process is simulated over the synthetic population described above, through a stochastic individual-based model that reflects the natural history of RSV infection. After birth, individuals are initially fully protected against RSV infection by the passive transfer of maternal-specific immunity. At each time step of the simulation, individuals protected by maternal antibodies become susceptible to the infection with probability *μΔt*, where *Δt* is the length of the time step (here, *Δt*=1 day) and 1/*μ* is the average duration of maternal protection, which is assumed to be 4 months [26]. Each susceptible individual can become infected through infectious contacts with household members, schoolmates (if attending a school), or through encounters in the general community. The latter accounts for all contacts not occurring within household or school settings, for example, at the workplace, on public transportation, at markets, and at shops. Specifically, at any time step *t* of the simulation, a susceptible individual *i* has a probability $\phantom {\dot {i}\!}p_{i}=1-e^{-\lambda _{i}(t) \Delta t}$ of being infected, where *λ*_*i*_(*t*) is the instantaneous risk of infection. We assume homogeneous mixing between all individuals who belong to the same setting, and thus the risk of infection *λ*_*i*_(*t*) can be computed for each individual *i* at any time step of the simulation as:

(1) $$\begin{array}{@{}rcl@{}} \begin{array}{lcl} \lambda_{i}(t) &=& \rho_{i} \beta \left(\frac{I^{h_{i}}(t)}{N^{h_{i}}(t)}+\frac{I^{s_{i}}(t)}{N^{s_{i}}(t)}+\frac{I(t)}{N(t)}\right) \end{array}  \end{array} $$

where:

- *i*,*h*_*i*_,*s*_*i*_ identify, respectively, an individual, his/her household, and (if any) his/her school;
- $\phantom {\dot {i}\!}I,I^{h_{i}},I^{s_{i}}$ represent the number of infectious individuals in the population, in household *h*_*i*_, and in school *s*_*i*_;
- $\phantom {\dot {i}\!}N, N^{h_{i}},N^{s_{i}}$ represent the number of individuals in the population, in household *h*_*i*_ and in school *s*_*i*_;
- *ρ*_*i*_ is the susceptibility to infection of individual *i*, which depends on the infection history of *i*; *ρ*=1 if *i* has never been infected and *ρ*=*x*<1 if *i* has already experienced an RSV infection;
- *β* is the setting-independent RSV transmission rate.

After each infection episode, infectious individuals recover with probability *γΔt*, where 1/*γ* is the average length of the infectious period, which is assumed to be 11 days, in agreement with the estimated average duration of shedding given in the literature [27]. Recovered individuals are initially fully protected against reinfection, but become susceptible (at least partially) again with probability *δΔt*, where 1/*δ* is the average duration of the temporary complete immunity against reinfection. At the beginning of the simulated epidemic, the fraction of individuals by age who had already experienced an RSV infection in the past was approximated by the age-specific fraction of seropositive individuals as observed in the cohorts followed between 2002 and 2005 [4]. Incidence rates of primary and repeated RSV infections by age were also derived from the same individuals and used to calibrate our model. Such approximations can be justified by the evidence that the RSV serological profile does not significantly change over a short period of time (from 2002 to 2005). Monthly importation of new RSV cases is considered in model simulations in order to account for a population that is not fully closed to RSV infections generated outside of the study area (see Additional file 1). Imported cases are randomly chosen in the population of susceptible resident individuals. The Kenyan school calendar year is considered in model simulations; that is, school enrollment is implemented at the beginning of January and school closures are implemented in April, August, and December (World Data on Education [28], accessed Jun 2014); RSV transmission in school is interrupted during these periods (that is, we assume $I_{s_{i}}(t)/N_{s_{i}}(t) = 0$).

We assume homogeneous mixing among individuals who share the same setting, and a setting-independent transmission rate. This means that: 1) each individual has contacts at random with all the individuals who share with her/him the same setting (for example, the same school or the same household); 2) the probability that a specific individual *i* has a contact with a specific individual *j* is inversely proportional to the setting size; 3) each contact between a susceptible and an infected individual generates a new infection with a probability that does not depend on the setting where the contact occurs.

A sensitivity analysis is reported in Additional file 1, where we assume three distinct transmission rates, one for each of the three distinct settings considered in the model: household, school, and general community.

It is shown that the set of strategies resulting in effective prevention of RSV infant infection does not change when the assumption of a setting-independent transmission rate is relaxed.

### Model calibration

The transmission model is calibrated by performing a Bayesian statistical analysis [29,30] of primary and repeated infection incidence data collected from a birth cohort of 635 children in Kilifi, Kenya, each of whom was followed over three consecutive RSV seasons (from 2002 to 2005) [3,4,31]. The model has the following free parameters: 1) the RSV transmission rate *β*; 2) the average duration of complete immunity generated from each infection event 1/*δ*; 3) the relative susceptibility to RSV reinfection once temporary immunity has waned *x*.

The posterior distributions of the free model parameters were explored by Markov chain Monte Carlo (MCMC) sampling applied to the likelihood of observing the age-specific RSV incidence, stratified by primary and repeated infections, in a population which reflects the Kilifi cohort study [4]. Specifically, by assuming that for each considered age group the number of observed RSV cases is binomial *B*(*n*,*p*), the likelihood is defined as:

$$\mathcal{L}=\prod_{j} \prod_{a} \binom {n(a)} {k_{j}(a)}p_{j}(a;\theta)^{k_{j}(a)}\left(1-p_{j}(a;\theta)\right)^{n(a)-k_{j}(a)} $$

where index *j* runs over the two types of infection (primary and repeated), index *a* runs over the age groups considered in the cohort study (0-5, 6-11, 12-17, 18-23, 24-30 months), *n*(*a*) is the number of individuals of age *a* at the beginning of the interval considered, *k*_*j*_(*a*) is the number of infections of type *j* reported at age *a*, and *p*_*j*_(*a*;*θ*) is the probability of type *j* infection for individuals of age *a* as predicted by the model simulations with parameter set *θ*=(*β*,*δ*,*x*). Notice that if some children acquire both primary and repeated infections at age *a*, both infection episodes are considered in the computation of *k* and *p*. Details on model calibration are reported in Additional file 1.

### Vaccination

Vaccinated individuals (including those who have already experienced RSV) are assumed to gain a temporary full protection against infection. Similarly to natural infection, once temporary vaccine protection has waned, immunized individuals are assumed to become susceptible to RSV infection.

Given the unknown efficacy of potential RSV vaccines and the uncertainties surrounding future coverage, we consider three scenarios on the percentage of successfully immunized individuals in the target population, namely, 100%, 80%, and 60%. Also, three different scenarios on mean duration of vaccine protection against RSV infection are evaluated: 4, 6, and 12 months.

We first considered a routine universal vaccination strategy at 3 months of age, with and without a catch-up campaign targeting individuals between 3 months and 15 years of age for the first year of the program. Immunization at 3 months of age is simulated in order to evaluate potential benefits resulting from the availability of a vaccine suitable for being administered to infants. Routine immunization of infants is assumed to occur within the third month of age of each child.

The second targeted age group was school-age children. Specifically, a one-off vaccination at first school enrollment is evaluated, with and without a catch-up campaign in the first year of the program targeting only students of primary schools. Potential effects of repeating the vaccination campaign annually, as recently implemented in the UK [32] for controlling seasonal influenza, are also explored by assuming all primary students as our annual target population. The effects of targeting only students cohabiting with infants are also explored. Vaccination at school enrollment, vaccination of all students, and all catch-up strategies are implemented on the first day of January.

Thirdly, in line with renewed interest in targeting pregnant women for RSV vaccination [14] (see also [33], accessed Jun 2014), we explored this strategy under two different assumptions: a) the vaccine has no direct protective effect on the offspring of immunized women who will benefit only indirectly from herd immunity effects; b) the vaccine administered to pregnant women provides a longer duration of maternal antibody protection against RSV infection to their newborns. In the latter case we assume that vaccination of pregnant women extends the duration of natural maternal protection provided by unvaccinated mothers (which is assumed to be 4 months) to 5, 6, and 8 months, representing, respectively, an additional 1, 2, and 4 months protection due to vaccine boosting. Vaccination of pregnant women is performed at each birth.

## Results and discussion

### RSV transmission in the Kilifi cohort: data and simulation

**Figure 1:**
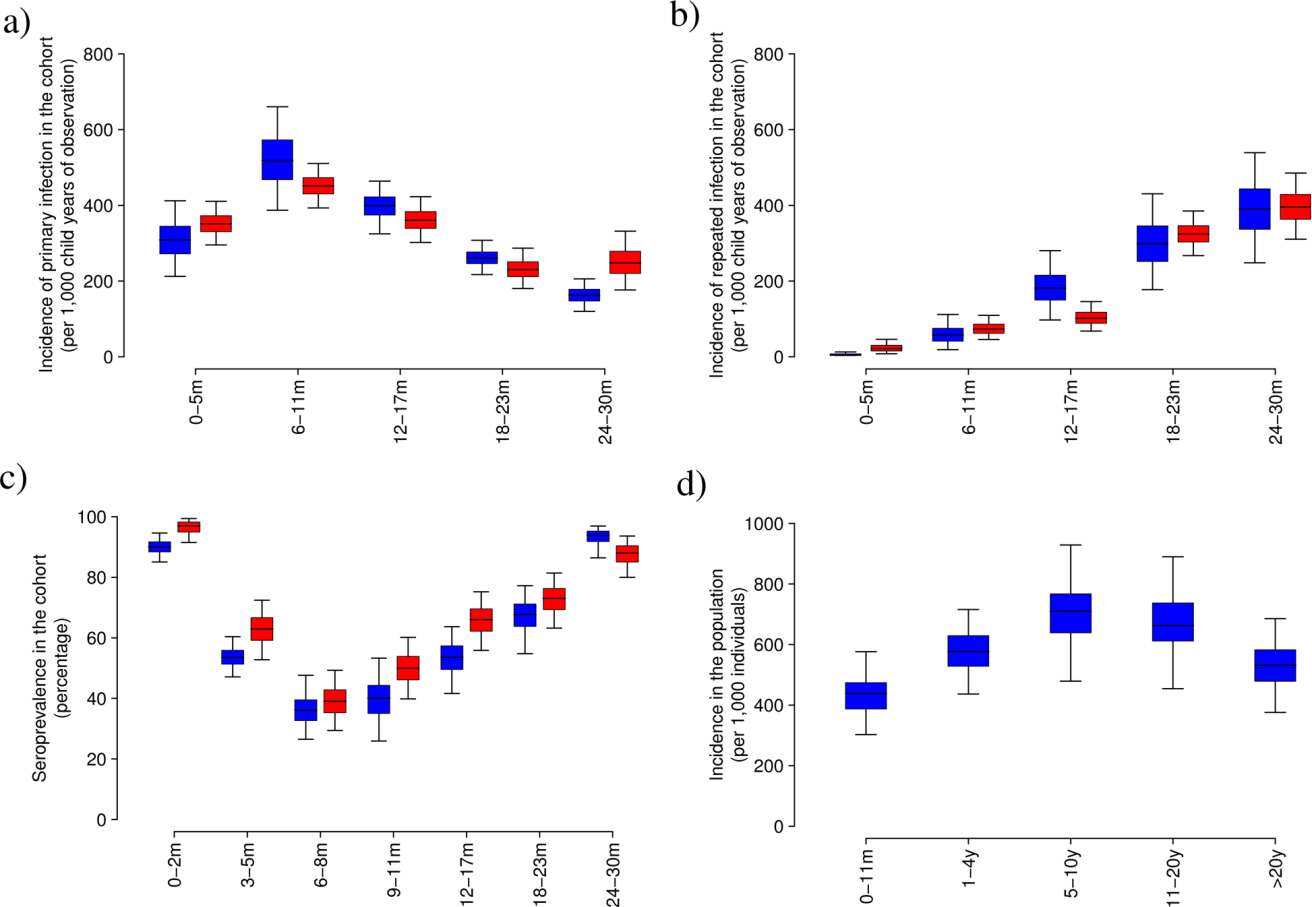
**RSV transmission patterns A.** Distribution (2.5%, 25%, 75%, and 97.5% quantile and mean) of incidence of primary **(a)** and repeated **(b)** RSV infection by age, simulated (blue) and observed (red) in a birth cohort of children followed for three RSV seasons in Kilifi, Kenya [4]. Data credible intervals were computed through exact binomial test. **(c)** Distribution (2.5%, 25%, 75%, and 97.5% quantile and mean) of simulated (blue) and observed (red) age-specific RSV IgG seroprevalence within the followed cohort after three RSV seasons [4]. **(d)** Distribution (2.5%, 25%, 75%, and 97.5% quantile and mean) of simulated incidence of RSV infection by age.

In agreement with previous findings [4], our estimates suggest that RSV infection generates a period of complete immunity to reinfection lasting, on average, 6.56 months (95% CI 1.80,14.7), after which little resistance to repeated infection remains (the relative susceptibility is estimated to be, on average, 0.88 95% CI 0.41,0.99). Model predictions on the age-specific incidence of primary and repeated RSV infection based on the estimated posterior distribution of the model parameters robustly capture the observed patterns (see Figure 1). In particular, our results show that primary incidence peaks before 12 months of age (see Figure 1a), with young infants (<6 months) suffering a lower risk of RSV infection mainly due to passive transfer of maternal-specific immunity [26,34]. Primary incidence decreases with age for children older than 1 year, for which a larger fraction of individuals has already experienced their first RSV infection episode (see Figure 1c). On the other hand, repeated RSV infection incidence among young children (<30 months) increases steadily with age (see Figure 1b). The predicted age-specific RSV serology, in line with the observed values, shows that almost all individuals have already experienced at least one RSV infection at 2 years of age (see Figure 1c); the estimated median age at first infection is, on average, 16.0 months (95% CI 12.5,20.8).

### RSV transmissibility potential

In order to provide useful insight on the transmissibility potential of RSV in the considered population a range of possible reproductive numbers based on the proposed transmission model and on the parameter estimates obtained by model calibration were computed.

In particular, we first compute the basic reproduction number *R*_0_ and the effective reproductive number *R*_*e*_, which are respectively defined as the average number of individuals infected by a typical infectious individual in a fully susceptible population and when a fraction of the population is - at least partially - protected against the infection. Second, the transmission potential of RSV infection has also been investigated by computing the *R*^*index*^ and the $R_{e}^{index}$ which are respectively defined as the average number of individuals infected by the first infectious individual (the index case) in a fully susceptible population and in a partially immune population. The procedures adopted for computing *R*_0_,*R*_*e*_,*R*^*index*^, and $R_{e}^{index}$ are detailed in Additional file 1.

**Figure 2:**
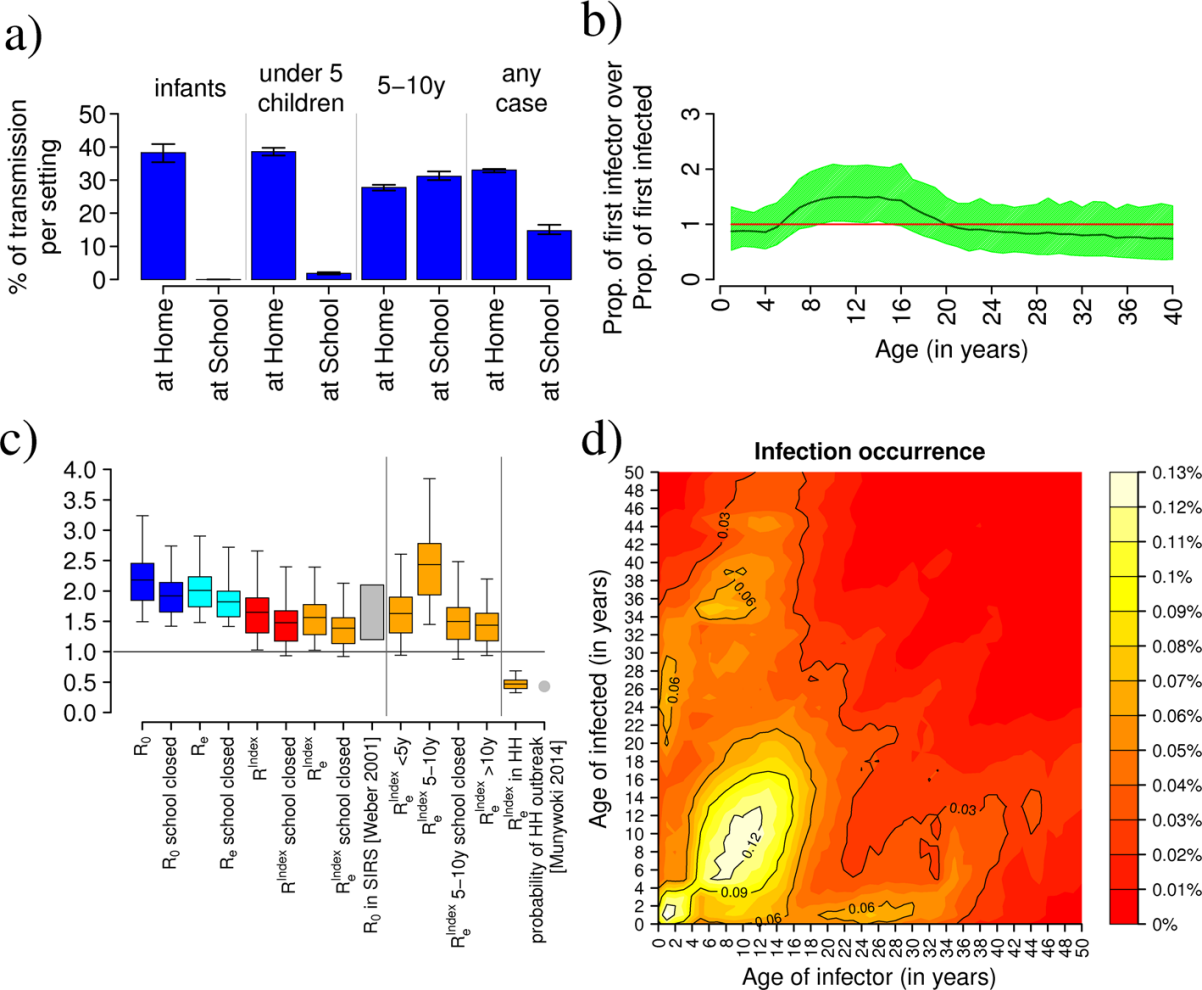
**RSV transmission patterns B.** **(a)** Simulated proportions of transmission occurring at home and at school stratified by age class. Vertical lines represent 95% credible intervals. **(b)** Simulated age-specific ratio between the probability of being an index case in a household outbreak over the probability of being the first secondary case generated by household transmission. Shaded area represents 95% credible intervals. **(c)** Transmissibility potential of RSV. **(d)** Percentage of infection episodes generated through household transmission due to contacts occurring between individuals of different ages.

Results are summarized in Figure 2c. We found that *R*_0_ is 2.18 (95% CI 1.49,3.24), *R*_*e*_ is 2.0 (95% CI 1.48,2.91), *R*^*index*^ is 1.65 (95% CI 1.03,2.66), and $R_{e}^{index}$ is 1.57 (95% CI 1.02,2.39). Values obtained for *R*^*index*^ and $R_{e}^{index}$ are lower than those for *R*_0_ and *R*_*e*_ as the initial infective individual is randomly chosen and therefore it cannot be considered a “typical” infector, as required in the definition of *R*_0_ and *R*_*e*_. The obtained difference between the estimated *R*_*e*_ and $R_{e}^{index}$ is in line with literature values [35,36] and estimates of *R*_0_ and *R*_*e*_ do not remarkably change when importation of new cases is not considered in model simulations.

The obtained estimates of the RSV transmission potential are in satisfactory agreement with independent estimates obtained elsewhere by using an SIRS model (that is, with no partial lifelong immunity) [24]. In addition, by computing the average number of individuals infected by the first infectious individual in a partially immune population within her/his household we inferred the chance of observing a household outbreak after the introduction of RSV in a household. Our results suggest that a randomly sampled index case produces on average 0.50 case (95% CI 0.41,0.55) within the household, therefore resulting in a household outbreak in 50% of cases. This estimate is in line with results obtained in [Munywoki et al. 2014] where out of 73 separate RSV introductions into households, only 32 (43%) generated a household outbreak.

Finally, estimates of *R*_0_, *R*_*e*_, *R*^*index*^, and $R_{e}^{index}$ are lower when no transmission in schools is assumed (for example, when schools are closed). This suggests that schools and students play a relevant role in the transmissibility of RSV in the population. This is supported by the investigation of $R_{e}^{index}$ stratified by age classes which highlight that the $R_{e}^{index}$ of students is 67% higher than the average $R_{e}^{index}$, but that it is reduced by 40% when schools are closed.

### The RSV transmission chain: the role played by households and school-age children

In agreement with a recent epidemiological study which investigated the role of household transmission in a rural community in the Kilifi district [15], we found that the percentage of infant primary infections generated by household transmission in a specific subgroup of households (see [15] for details) is on average 49.5% (CI 95% 41.0,58.5) (versus 53% in [15]). Such results refer to a set of households chosen among those with at least one infant and one or more older siblings under 13 years of age [15].

By extending our analysis to all types of households and by accounting for both primary and repeated infections, we found that, on average, 38.3% (CI 95% 35.4,40.9) of infants and 38.6% (95% CI 37.4,40.0) of children under 5 years of age contract RSV as a consequence of contacts occurring within households. On the other hand, among older children (5-10 years), where school attendance rates are high (see Additional file 1), school transmission is predicted to be responsible for about 30% of RSV infections (see Figure 2a).

The contribution of household contacts to overall RSV transmission (that is, irrespective of age) increases with household size. For instance, while 33.0% (95% CI 32.4,33.4) of transmission in the overall population occurs in the household, if we restrict our analysis to households of two individuals, the resulting average proportion of cases generated within the household is less than 12%, while it is about 35% when considering households with ten individuals or more (details are reported in Additional file 1).

Our analysis allows the identification of individuals who introduce the infection into the households (index cases) as well as the first cases generated by household contacts (secondary cases). We found that, on average, 54.6% (CI 95% 41.6,65.8) of index cases responsible for a household outbreak were school-age children. Moreover, the role played by different age categories in introducing RSV into households was highlighted by computing for each age the ratio between the probability of being an index case and the probability of being the first secondary case generated by household transmission. The resultant ratio is presented in Figure 2b and shows that school-age children are, on average, up to 50% more likely to be index cases than first secondary cases.

We further investigated household transmission by recording, for each case generated within households, both the age of the infected individual and the age of its infector. Figure 2d aims to highlight where (in terms of ages) most of the infections occur by showing the proportion of incidence of within household transmission due to contacts occurring between individuals of different ages. Specifically, in Figure 2d matrix *M*_*i*,*j*_ is reported, where $M_{i,j} = 100 \cdot C_{i,j} /\sum _{i}\sum _{j} C_{i,j}$ and *C*_*i*,*j*_ is the recorded number of household infection episodes where an individual of age *i* was infected by an individual of age *j*.

Our results show that most of the transmission occurs between siblings of similar age and between parents and their children. However, school-age children are predicted to be the main source of household transmission, causing approximately 55% of all RSV cases generated within household and 40% of infant cases. This indicates that vaccination of school-age children might represent a valuable strategy for decreasing the occurrence of infection within households and, in turn, among infants.

### Preventing RSV in infants with short-lived vaccine immunity

**Figure 3:**
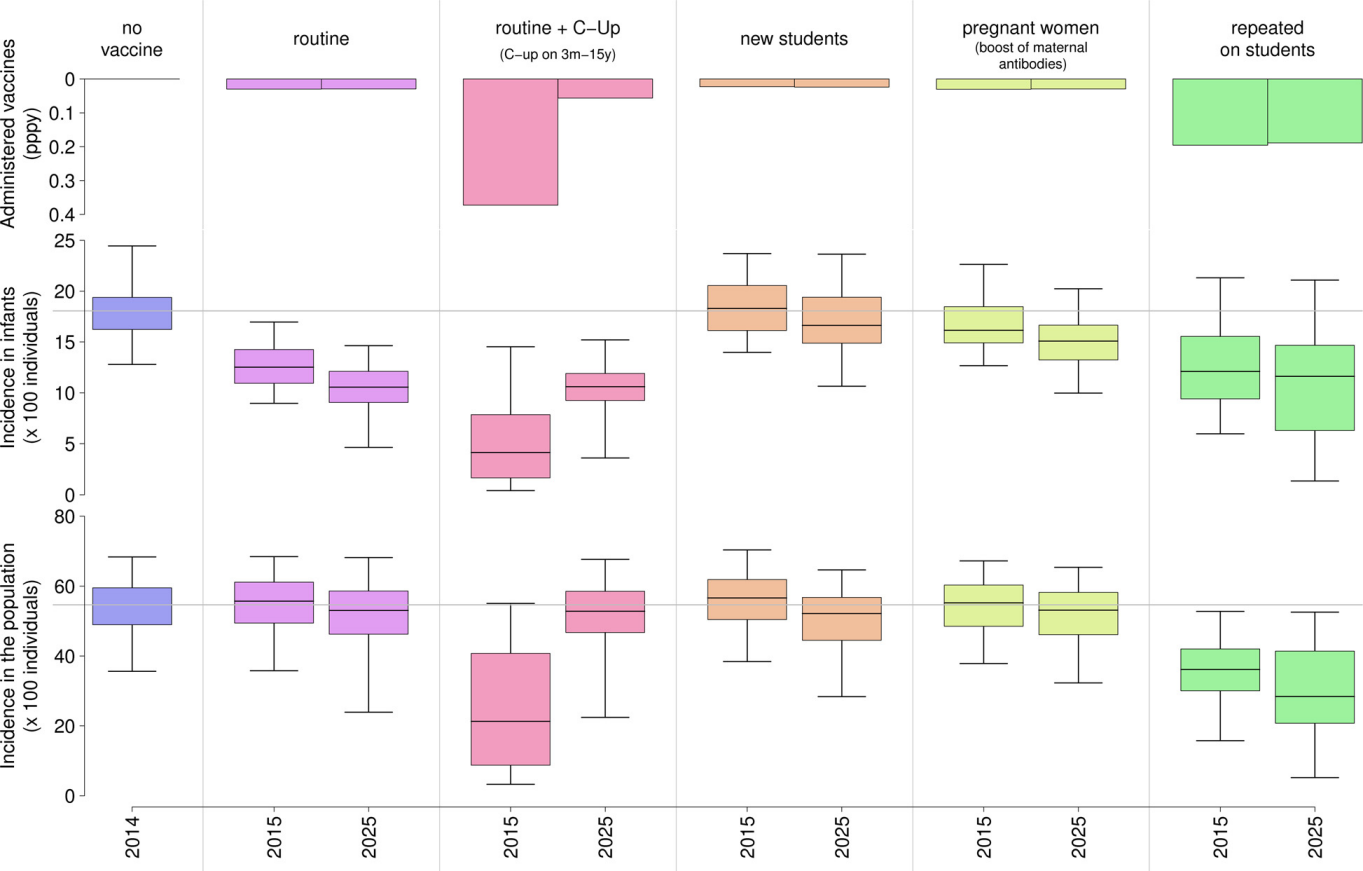
**Impact of vaccination strategies: baseline scenario.** Average number of administered vaccine per person per year (top row), distribution (2.5%, 25%, 75%, and 97.5% quantile and mean) of RSV incidence in infants (middle row), and in the general population (bottom row) as predicted by model simulation before vaccination (year 2014) and 1 and 10 years after vaccination (years 2015 and 2025) associated with the most effective vaccination strategies. Vaccine protection against infection is assumed to last 6 months and vaccination coverage is 100%. Vaccination of pregnant women is assumed to extend natural maternal protection of newborns from 4 to 6 months. The gray line reported throughout is used as a reference indicator for the no vaccination scenario.

The impact of different immunization strategies was evaluated in terms of predicted reduction of the infection incidence among infants and in the general population, the number of vaccine doses administered, and the age at first infection. These outcomes have been analyzed 1 and 10 years from the start of vaccination. In Figure 3 the estimated impact of the most effective vaccination strategies is shown assuming a vaccination coverage of 100% and a vaccine protection that lasts 6 months, which reflects the estimated duration of immunity provided by natural infection (the impact of the remaining strategies is shown in Additional file 1).

Our results show that routine immunization at 3 months of age and annual repeated vaccination of all students are the only two strategies able to trigger a relevant and persistent reduction of RSV infection occurrence in infants. Specifically, in 10 years of vaccination, the former strategy is predicted to reduce RSV infant infection incidence by 41.5% (95% CI 40.2,63.8) with little impact on the other age groups, while the latter strategy is predicted to generate a reduction of 35.6% (95% CI 13.7,89.4) in infants and 48.0% (95% CI 23.1,85.4) in the general population. The benefits resulting from catch-up campaigns performed in the first year of vaccination completely wane after a few years (less than five), and require a significant increase in the number of administered vaccines (see Figure 3 and Additional file 1).

Although repeated vaccination of students entails a relatively high number of annually administered vaccines, namely 0.19 dose per person per year, this strategy is expected to be the most valuable alternative to infant immunization if RSV vaccines would not be suitable to be administered safely to infants. In addition, by employing this immunization strategy, the median age at first infection is expected to increase significantly, from 16.0 months to 21.0 months on average (see Additional file 1), possibly leading to a reduction in the incidence of severe disease.

It is worth noting that vaccination targeting only students (or new students) cohabiting with infants is not enough to break the RSV transmission chain leading to infant infection, entailing only negligible delays in the median age at first infection (see Additional file 1).

### The potential benefits of longer vaccine immunity

**Figure 4:**
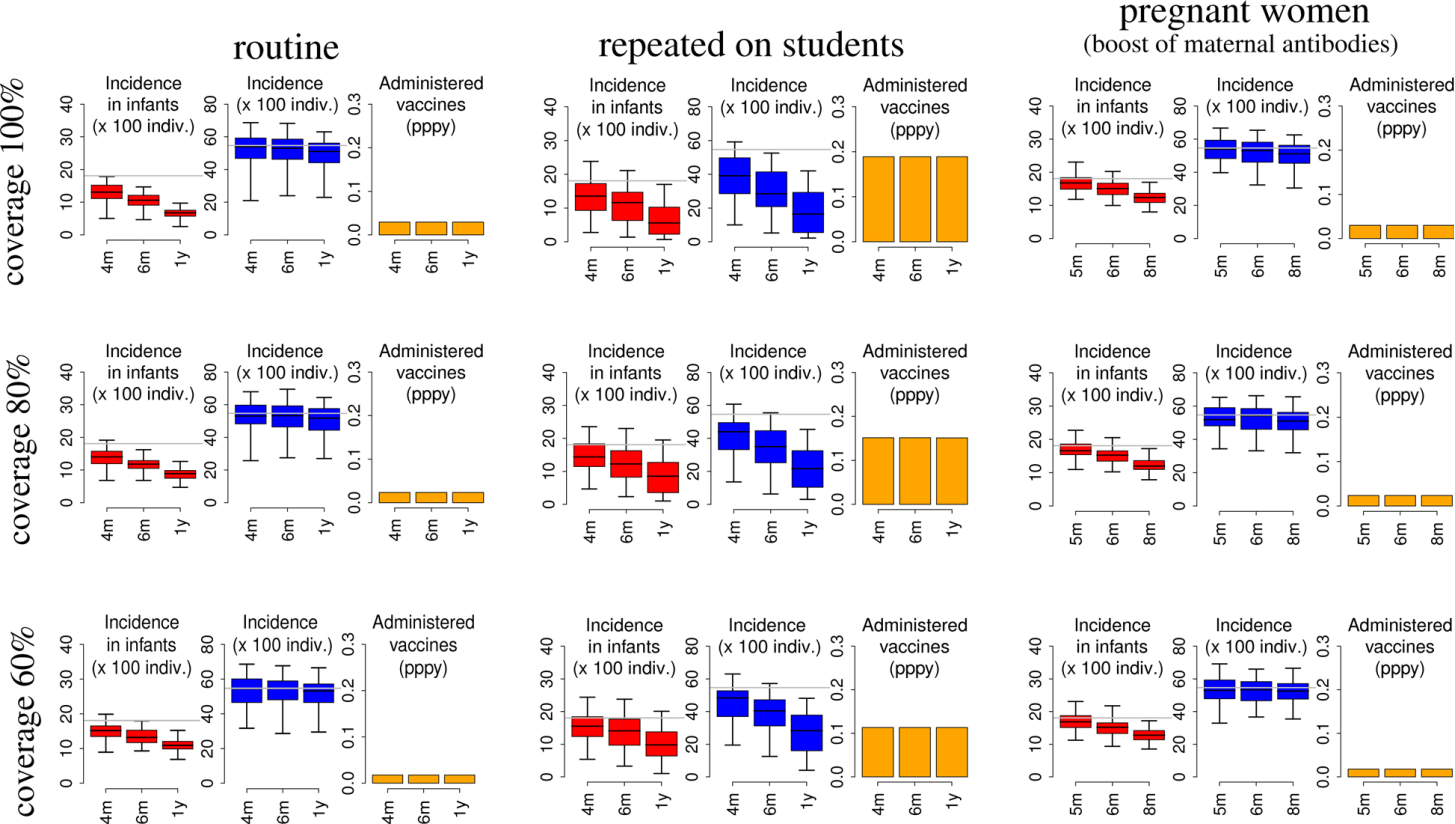
**Impact of vaccination strategies: alternative scenarios.** Distribution (2.5%, 25%, 75%, and 97.5% quantile and mean) of RSV incidence in infants (red) and in the general population (blue) and the average number of administered vaccines per person per year (orange) as predicted by model simulation after 10 years of vaccination for 3 selected strategies under different assumptions on the duration of vaccine protection (4 months, 6 months, and 1 year) and on the percentage of successfully immunized individuals in the target population (100%, 80%, and 60%). Gray lines reported in the plots represent the average value predicted for the no vaccination scenario. In the scenario in which vaccine protection of newborns results from vaccination of pregnant women, 5, 6, and 8 months duration represent extended durations due to vaccine boosting of 1, 2, and 4 months, respectively.

The estimated impact of different immunization strategies after 10 years of vaccination under different assumptions on the duration of vaccine-induced immunity and on the fraction of successfully immunized individuals in the target population is summarized in Figure 4. Our results show that if the vaccine can induce longer protection than natural infection, all the considered strategies perform better, and some additional immunization strategies have the potential to remarkably reduce RSV infant infections. Specifically, when the administration of a vaccine to pregnant women is able to boost natural maternal antibodies to 8 months of protection against RSV infection in their offspring (extending maternal protection by 4 additional months), the strategy of targeting future mothers can be considered an additional and valuable alternative to the direct immunization of infants, resulting in a reduction in the RSV infant incidence of 31.5% (95% CI 30.7,37.5). In contrast, if newborns will benefit only from the (indirect) herd immunity effect, immunization of pregnant women is not expected to prevent RSV infection in infants (see Additional file 1). In comparison, when vaccine-induced immunity lasts 1 year, the routine immunization at 3 months of age and the annual repeated vaccination of all students can be considered similarly effective, leading to a reduction in RSV infant incidence ranging, on average, from 62.9% to 69.1%.

Unfortunately, for a vaccine-induced immunity shorter than the protection provided by natural infection (for example, 4 months), the ability of the latter strategies to prevent RSV infection in infancy is markedly reduced (see Figure 4). Indeed, in this case, the expected average reduction of RSV infant incidence after 10 years of vaccination with routine immunization and annual vaccination of students ranges between 23.4% and 24.2%, on average.

### The scenario of sub-optimal coverage

If the fraction of successfully immunized individuals in the target population is reduced - as a consequence of either a lower vaccine coverage or a limited vaccine efficacy - the expected reduction in RSV incidence among infants and in the general population achieved after 10 years of vaccination decreases for all the considered strategies (see Figure 4). However, if the vaccine-induced immunity lasts 1 year, even when only 60% of the targeted population is protected, routine immunization of infants and repeated vaccination of all students are predicted to reduce infant RSV incidence by on average 39.6% and 45.6%, respectively, after 10 years of vaccination.

In contrast, the effectiveness of newborn immunization through vaccination of pregnant women seems to be only slightly affected by sub-optimal coverage. Indeed, under all assumptions on the duration of vaccine-induced immunity, when the fraction of protected infants through vaccination of pregnant women is 60%, the reduction of RSV infections in infants is predicted to be at most 4% lower than what could be achieved with 100% coverage and a fully effective vaccine. The robustness of this strategy is especially relevant considering that the delivery of a vaccine through antenatal care may be sub-optimal because of possibly low women attendance rates to antenatal clinics, and also in terms of protection because of premature births. This means that, if a vaccine able to significantly boost maternal protection of newborns through vaccination of pregnant women would be available, this strategy might possibly represent the best trade-off between the ability of preventing RSV in infants and a limited number of doses required.

## Conclusions

Although vaccine development aimed at prevention of RSV disease in young children dates back to the 1960s, and several trials are ongoing to identify a suitable candidate product, no vaccine has been licensed yet [14]. A better understanding of the RSV transmission chain leading to infant infection can be critical to the selection of suitable candidate vaccines and population targets [14,37].

With this objective in mind, we have developed an individual-based model for investigating RSV transmission patterns in a low-income setting of rural coastal Kenya. The chosen modeling framework has allowed us to disentangle the contribution of different social settings in the spread of the infection in the population, to characterize the transmission chain leading to RSV infant infections, and to investigate the impact of a range of immunization schedules that are plausible alternatives to routine early infant vaccination. Specifically, these options include vaccination of school children, siblings of susceptible naive infants, and pregnant women.

Our analysis determined household transmission to be responsible, on average, for about 39% of infant infections and found that school-age children play a key role in introducing RSV infection into the household, causing about 55% of household outbreaks. Moreover, our results showed that RSV infection provides complete natural immunity against reinfection for a period of around 6 months, after which resistance to reinfection appears very limited.

The vaccination strategies considered are shown to reduce RSV infection incidence both in infants and in the general population in different ways, and their impact critically depends on the duration of vaccine-induced protection. However, model predictions robustly suggest that as an alternative to direct vaccination of very young children, prevention of RSV in infancy can be achieved by reducing the transmission in the general population through the vaccination of students. In particular, in the case of a short-lived vaccine-induced immunity, for example, 6 months, the annual vaccination of all students is predicted to be the most valuable alternative to the direct immunization of young infants, reducing RSV infection occurrence in those less than 1 year of age by more than 35% after 10 years of vaccination, as opposed to a 41% reduction of infant infections when directly targeting 3-month-old babies. Additionally, a maternal vaccine able to increase the average duration of passive RSV protection by only 4 months represents an effective strategy for preventing RSV occurrence in infancy, even in the case of sub-optimal coverage. In particular, in this case, our results show that RSV infant infection incidence could be reduced through vaccination of pregnant women by at least 30%.

In conclusion, our results robustly show that school-age children should be considered for alternative effective vaccination programs in case direct immunization of high-risk infants is not achievable. In addition, vaccination of pregnant women also has the potential of being an effective strategy. Clearly, for strategies targeting the transmitters of infection rather than those most vulnerable to disease following infection, education of the public will be a priority to ensure the acceptability and sustainability of such immunization programs [32,38].

Finally, note that in this work there is no attempt to quantify the impact of vaccination on the risk of disease following infection. Since the changes in incidence predicted are accompanied by changes in the age distribution of infection (to a degree varying by strategy), and because the risk of disease is highly dependent on age at infection, then the outcomes of vaccine impact on infection and on disease are not directly proportional. Thus, for example, the effect of repeated vaccination to school age children with large effect on age at infection would result in a proportionally higher effect on RSV disease. Also, the effect of maternal vaccination and increased duration of passive immunity in infants would directly protect those most at risk of severe disease, and so the impact on disease would be more significant than the reported impact on infections.

## Additional file

Additional file 1
**Supporting material.** Model details, additional results.
