# Supplementary material for: Evaluating vaccination strategies for reducing infant respiratory syncytial virus infection in low-income settings
Source: BMC Med. 2015 Mar 10;13:49. doi: 10.1186/s12916-015-0283-x (PMC4372170; doi:10.1186/s12916-015-0283-x)
Supplement: Additional file 1 — Supporting material. Model details, additional results. [file 12916_2015_283_MOESM1_ESM.pdf]

Evaluating vaccination strategies for reducing infant respiratory syncytial virus infection in low-income settings

Piero Poletti[1,2], Stefano Merler[2], Marco Ajelli[2], Piero Manfredi[3], Patrick K. Munywoki[4], D. James Nokes[4,5] & Alessia Melegaro[1]

[1] Dondena Centre for Research on Social Dynamics, Universitá Commerciale L. Bocconi, Milan, Italy
[2] Center for Information Technology, Bruno Kessler Foundation, Trento, Italy
[3] Department of Statistics and Mathematics Applied to Economics, University of Pisa, Italy
[4] Kenyan Medical Research Institute (KEMRI) Wellcome Trust Research Programme, Centre for Geographic Medicine Research – Coast, Kilifi, Kenya
[5] School of Life Sciences and WIDER, University of Warwick, UK

Supplementary Material

Contents

1 Materials and Methods **2**
1.1 Empirical data 2
1.2 Socio-demographic model 2
1.3 Additional details on the RSV transmission model 6
1.4 Parameters estimation 7
1.5 RSV transmissibility potential 9

2 Additional results **9**
2.1 The role played by household size 10
2.2 Age at first RSV infection 11

3 RSV infection incidence in adults **12**

4 Sensitivity analysis on transmission rates by settings **12**

Bibliography **22**

1 Materials and Methods

The demographic model is characterised by a highly detailed social structure, which incorporates households and schools. This is a critical component of the framework used as it allows to consider the present heterogeneities in contact patterns among individuals. In addition, the explicit representation of each individual in the population allows us to simulate control strategies targeting individuals instead of class of individuals, such as the immunization of siblings of susceptible naive infants and the vaccine administration to pregnant women.

1.1 Empirical data

The socio-demographic structure of the model, both at the individual and at the household level, is generated using the Demographic and Health Survey data for Kenya collected in 2003 [1]. In particular, the following variables were considered:

- household records: age of the household head, list of household members characterized by their age, sex and relationship with the household head
- individual records: age, marital status, number of children, school enrollment of a) women between 15 and 50 years of age and b) men between 20 and 55 years of age; age and school enrollment for children younger than 15 years of age;
- birth records: date of birth, age of mother and number of siblings at birth.

Two data sources were used to generate schools and to assign children to schools: 1) school size distribution of primary schools taken from the Open Kenya data set [2]; 2) school attendance rates by age taken from the DHS individual records.

The RSV transmission components of the model were calibrated using data from a cohort study carried out between 2002 and 2005 in the Kilifi district during 3 consecutive epidemics [3]. In this study the incidences of primary and repeated RSV infections are reported for the following age groups: 0-5m, 6-11m, 12-17m, 18-23m 24-30m. The reported serological profile obtained at the end of the study period in the considered cohort is instead provided for the following age groups: 0-2m, 3-5m, 5-8m, 9-11m, 12-17m, 18-23m, >24m.

1.2 Socio-demographic model

Each individual i in the model is characterized by: age, sex, the index of the household where she/he lives in h_i and, if individual i is a student, the index of the school she/he attends s_i. A schematic diagram of how individuals are considered in the model is shown in Fig. S1.

Generation of individuals and households

The synthetic population of individuals is generated iteratively, household by household, according to a stochastic procedure that starts from the generation of the head of the

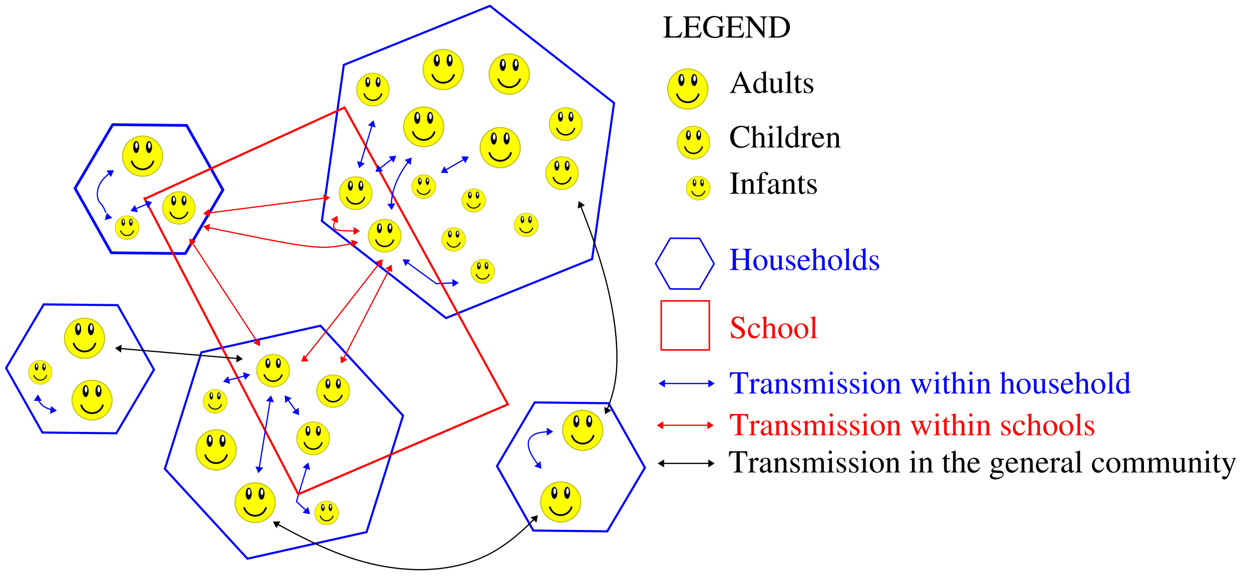

Figure S1: Schematic representation of the model.

household, then followed by the generation of her/his relatives. Different household structures and sizes are obtained indirectly by generating step by step individuals associated to the household head and by co-locating them in her/his household.

Specifically, the first member of a household is generated and her/his age a_h and marital status m_h are assigned according to the age distribution of household heads and frequencies of married, widowed, divorced and never married household heads of age a_h, as resulting from the analysis of the DHS household records.

Additional household members are generated according to the probability that a household head of age a_h is not living alone and according to:

- the probability $P(w|a_h, m_h)$ of observing w wives/husbands of the household head in an household having head of age a_h and with marital status m_h, stratified by sex of the household head;
- the probability $P(b|a_h)$ of observing b brothers of the household head in a household having head of age a_h;
- the probability $P(s|a_h)$ of observing s sisters of the household head in a household having head of age a_h;
- the probability $P(p|a_h)$ of observing p parents/parents-in-law of the household head in a household having head of age a_h;
- the probability $P(c_h|a_h, m)$ of observing c_h sons/daughters of the household head h, given household head age a_h and marital status m_h.

It is worth noting that the model accounts for the possibility of multiple partners, but only in the case of the household head. Nonetheless, the occurrence of multiple partners

observed in the dataset used is negligible, so in more than 99% of cases the number of partners is equal to 1.

The age of each generated household member a_m is assigned by using the distribution of the age difference, as reported in the DHS records, between the household head h and the "type" t_m (defined by her/his relation with the household head) of individual m, stratified by the age of household head. In addition, gender is assigned to each new generated individual by considering a 1:1 sex ratio.

In order to account for the co-location of up to 4 generations within a single household (from parents of the household head to her/his grandchildren) and to reproduce the complex structure observed in Kenyan households (e.g., the presence of daughter/son in law of the household head), we employed the following procedure repeated over each household member m generated at the previous step, except for the household head and her/his possible partner:

- given the type t_m of household member m (defined by her/his relation with the household head) and her/his age a_m, the marital status m_m is assigned according to the probability $P(m_m|a_m, t_m)$ of observing the marital status m_m in individuals of age a_m and type t_m in the DHS household records;

- partners of married members are generated and assigned to the household, while their age is assigned according to the distribution of the age difference between husband and wife reported in the DHS records and stratified in 10 age classes of the husband;

- for each generated women w of age a_w, marital status m_w and type t_w, which is defined as her relationship with the household head (e.g. sister, daughter, daughter in law, etc.), c_w children are assigned to the household according to the observed probability $P(c_w|a_w, m_w, t_w)$ of having c_w sons or colorblack daughters living in the same household of their mother among mothers of age a_w, marital status m_w and type t_w.

- the age of new generated children (i.e. of nephews, nieces and grandchildren) is assigned according to the distribution of the observed age difference between mothers and children, computed conditionally to the cardinality of the child considered (1st child, 2nd child, etc.); gender is assigned to new generated children by considering a 1:1 sex ratio;

At the end of the above described procedure, households are defined as the set of individuals associated to a specific household head (including the houshold head). Individuals are thus characterized by age, sex, marital status and their relation with the household head (wife, sister/brother, son/daughter, son/daughter in law, grandchild, parent/parent in law, etc). The procedure is repeated until all individuals of the population are generated.

Generation of schools

Primary schools are generated according to the following procedure:

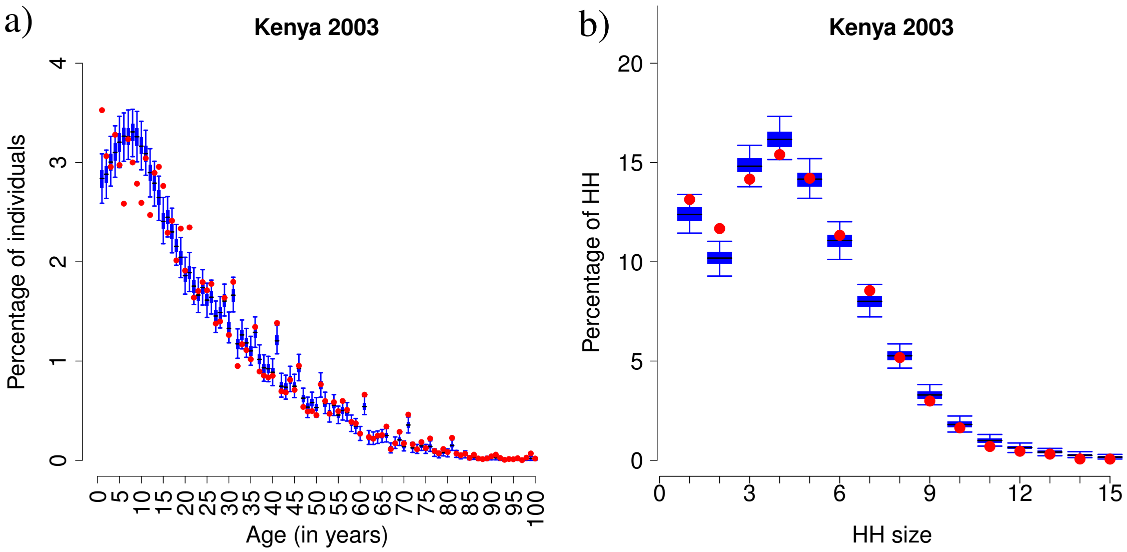

Figure S2: Validation of socio-demographic model. a) Boxplot (2.5%, 25%, 75% and 97.5% quantile and mean) of the age distribution of the population as simulated in the model (blue) and as computed according to DHS records (red); b) Distribution of household size in the population as simulated in the model (blue) and as computed according to DHS records (red).

- individuals are defined as students according to the age-specific enrollment rate reported in the DHS data-set;
- a set of schools is generated in such a way that each student of the synthetic population can find place in a school;
- the size of the generated schools is determined by randomly sampling from the distribution of school size reported in the Open Kenya data-set;
- students are randomly assigned to different schools.

Births, deaths, aging and school enrollment

The model accounts for the vital dynamics of the population. In particular, newborns are generated according to observed fertility rates as computed by using DHS birth records [1]; individuals die according to the observed mortality rate by age and gender, as reported in the World Population Prospects of the United Nations [4]. In the model births and deaths occur daily while the progressive aging of individuals occurs monthly. Moreover, once a year, in January, individuals can enter and leave schools, according to age-specific school enrollment rates as computed from DHS individual records.

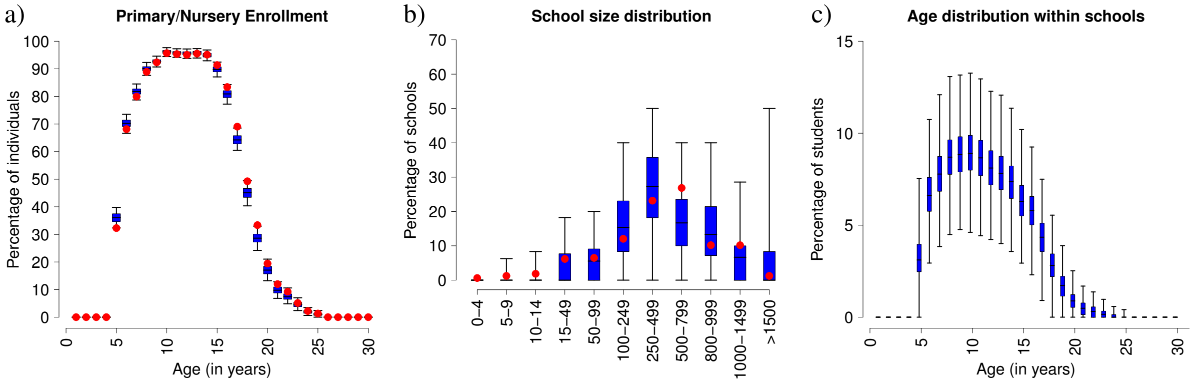

Figure S3: a) Boxplot (2.5%, 25%, 75% and 97.5% quantile and mean) of school enrollment by age for primary schools in Kenya as simulated in the model (blue) and as computed according to DHS records (red); b) Primary school size distribution in Kenya as simulated in the model (blue) and as computed according to Open Kenya (red); c) Simulated age distribution within schools.

Validation of socio demographic model

Model validation is carried out by running the algorithm described above for the generation of a synthetic population of about 25,000 individuals which reflects the size of DHS records (all the remaining simulations in the manuscript assume instead a population of 200,000 individuals). Repeated runs have been considered in order to account for the stochastic variability of the algorithm employed for households and schools generation.

The obtained results have been compared to DHS household and individuals records for the population of Kenya, in terms of age structure of the population and household size distribution, which represent two descriptive statistics of the modeled population that have not been directly used in the model to generate households structures and individuals' age.

As shown in Fig.S2 the generated synthetic population is compliant with descriptive statistics obtained from DHS records. The model is also able to well reproduce the age-specific school attendance rate computed from the DHS and school size distribution reported by Open Kenya (see Fig.S3).

1.3 Additional details on the RSV transmission model

Each simulated epidemic is initialized with 10 infected individuals randomly chosen in a synthetic population of 200,000 individuals and it runs for 4 consecutive years (i.e. from the beginning of 2002 to the end of 2005). The system is initialized with no other infections in the residual population and temporary protection against RSV infection gained by individuals during previous RSV epidemic seasons is assumed to have already waned. Individuals older that 5 years are assumed to have already experienced at least one RSV infection in the past, while only a fraction of individuals under 5 years of age is assumed to have already experienced RSV in the past. This fraction is set in order to reflect the RSV age-specific serological profile as observed at the end of the Kilifi cohort study [3]. Finally,

a fraction of individuals is assumed to be protected by maternal antibodies (M) according to the assumption that maternal protection lasts on average 4 months and that its duration is exponentially distributed. Infection is continuously sustained in the simulated epidemics by the importation of 5 new infected individuals each month. The individualbased model implemented in the manuscript is a discrete-time stochastic model and the time-step (i.e. $\Delta t = 1$day) was chosen in such a way that very few events per setting occur within each time step.

Model outputs obtained under different assumptions on the size of the population and on the number of monthly imported RSV cases have been compared in terms of RSV primary and repeated incidence by age, age-specific serological profile and percentage of transmission occurring in different settings. Results obtained by simulating a population of 20,000 and 500,000 individuals and obtained by considering a number of monthly imported cases ranging from 2 to 10 individuals have revealed that model predictions are not sensitive to different assumptions on these two factors.

1.4 Parameters estimation

Estimation of the free epidemiological parameters is performed by using a Bayesian Markov chain Monte Carlo (MCMC) method, implemented following a random-walk Metropolis-Hastings algorithm [5].

The method is used to estimate 1) the RSV transmission rate β, 2) the duration of complete immunity that generates from each infection event δ and 3) the relative susceptibility to RSV reinfection once temporary immunity has waned x.

Specifically, given the likelihood function $\mathcal{L}$ described in the main text, the Markov chain is generated in such a way that its stationary distribution represents the posterior distribution of parameters given the observed data. The chain is initialized with parameters drawn from uninformative uniform prior distributions as follows: $\beta = U[0, 1]$ days^{-1}, $\delta = U[0, 1000]$ days^{-1}, $x = U[0, 1]$. At each iteration, if the current value of the parameter is θ, a new value $\theta^\star = \theta e^{\sigma_\theta u}$ is generated, where u is drawn from a normal distribution $\mathcal{N}(0, 1)$ and σ_θ was adjusted in order to obtain an acceptance rate (percentage of accepted trials) close to 23% [6, 7, 8]. Since all the parameters are positive, a log scale was used to generate new parameters' values. The adopted procedure is equivalent to sampling the logarithm of the parameter θ from a Gaussian distribution (i.e. $log\theta^\star \sim \mathcal{N}(log\theta, \sigma_\theta^2)$, see [7, 8] for further details). Moreover, as the relative susceptibility to reinfection x is assumed to be a real number between 0 and 1, new values for x were generated by defining $x = 1/(1 + y)$, by generating $y^\star = ye^{\sigma u}$ and setting $x^\star = 1/(1 + y^\star)$.

Following the procedure already used in [9], new generated values $\theta^\star$ are accepted or rejected according to Metropolis-Hastings algorithm where the likelihood is approximated by the likelihood associated to a single realization of the parameter vector $\theta^\star$. The likelihood associated to the current value of the parameters θ is re-evalutaed at each step in order to ensure that the chain is not trapped at a local maximum due to stochastic variations on outputs associated to θ [9].

We performed 100,000 iterations and considered a burn-in period of 30,000 steps.

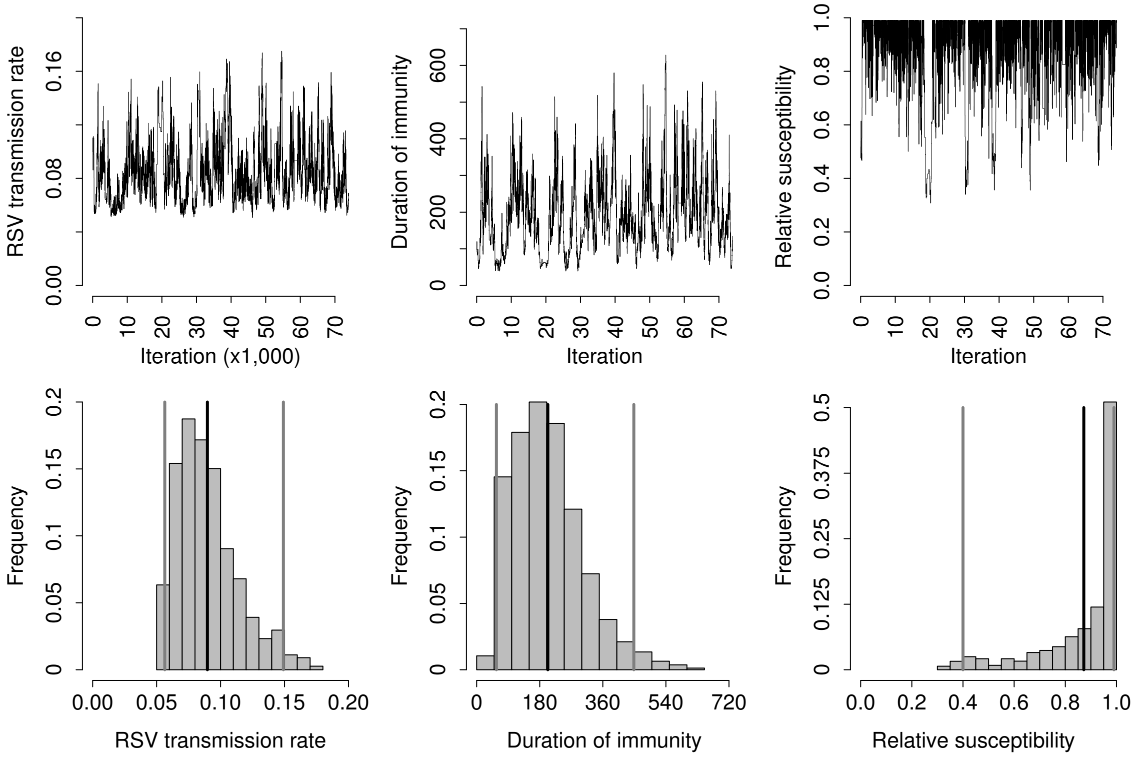

Figure S4: a,b,c) Trace plots of the MCMC chain used for estimating the posterior distribution of free parameters, respectively for the RSV transmission rate (β),the duration of temporary complete immunity (δ) and the relative susceptibility (x). d,e,f) Posterior distribution of parameters as obtained by the MCMC random-walk Metropolis-Hastings algorithm, respectively for β, δ, x. Vertical lines show mean values and the 95% of credible intervals.

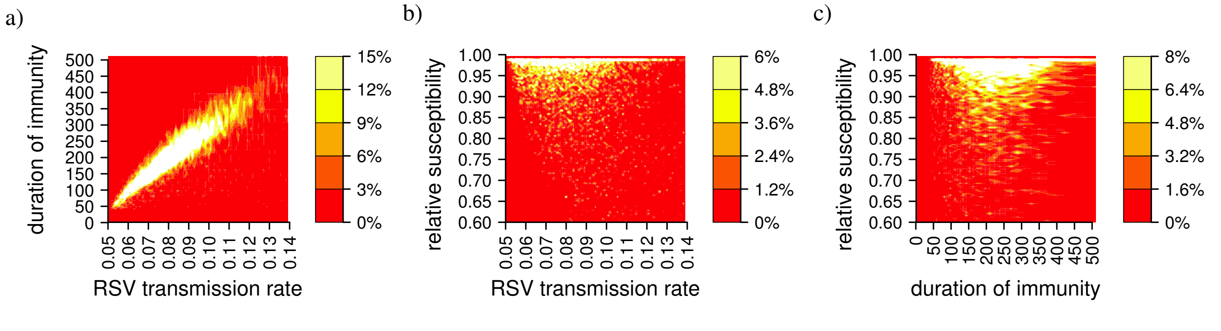

Figure S5: a,b,c) Joint pairwise posterior distributions of model parameters.

We checked convergence by considering chains associated to different starting points in the parameter space and by visual inspection on the trace plots of chains (see for instance Fig.S4a,b,c). The obtained posterior distributions of free parameters are shown in Fig.S4d,e,f.

As expected, a relevant correlation between the transmission rate and the average du-

ration of immunity has been found (see the joint pairwise posterior distribution in Fig.S5). Indeed, similar incidence rates can be obtained when either a shorter duration of complete protection gained after the infection is combined with a lower RSV transmission rate or a larger duration of complete protection gained after the infection is combined with a higher RSV transmission rate.

Finally, from the MCMC samples 1) we estimated the posterior means and 95% of credible intervals (CI) of the free parameters; 2) we sampled 1,000 parameters configurations that we use to investigate the RSV transmission patterns in the population, to disentangle the contribution of different social settings in the spread of the infection and to simulate the different vaccination strategies considered.

It is worth of noting that, under the assumption of a setting independent transmission rate, the contribution of different settings in the transmission of RSV is driven by a) the different mixing by age characterizing different settings (e.g., more assortative at school), b) the heterogeneity of mixing driven by the setting size (e.g., entailing repeated contacts with the same individuals in household and more dispersed when considering contacts occurring in the general community) and c) the heterogeneity between settings of the same type (e.g., household of different size and composition).

1.5 RSV transmissibility potential

Given that an explicit equation to compute the basic reproduction number for individual based models is not available, we inferred R_0 - as already proposed in the literature [10, 11, 12] - from the simulated epidemics. In particular, we defined R_0 =(1+rT) where T is the generation time, here defined as the average length of the infectious period, and r is the exponential growth rate estimated by fitting a linear model to the logarithm of the simulated infection incidence over time. It is worth noting that this simple formula, which is valid for simple homogeneously mixing SIR models, can be used to describe what happens in the initial phase of an epidemic driven by an SIRS model with reduced susceptibility. Indeed, given that the duration of temporary full protection is estimated to be 6 months on average, the SIRS model, at least at the beginning of the epidemic season, can be approximated by an SIR model when a fully susceptible population is initially considered. We apply the same technique to estimate the effective reproduction number (R_e), but by considering that a fraction of the population is partially immune. In this latter case, we assumed that individuals who have already experienced RSV in the past (i.e. a fraction by age that reflects the observed age-specific RSV seroprevalence) have a lower susceptibility to RSV infection (estimated through the MCMC procedure).

Finally, in the model, the R^{index} is computed by randomly choosing an index case among all susceptible individuals in the population and then keeping trace of all infections she/he is responsible for. A fraction of the population is again assumed to be partially immune to the infection at the beginning of the epidemic season when R_e^{index} is computed.

2 Additional results

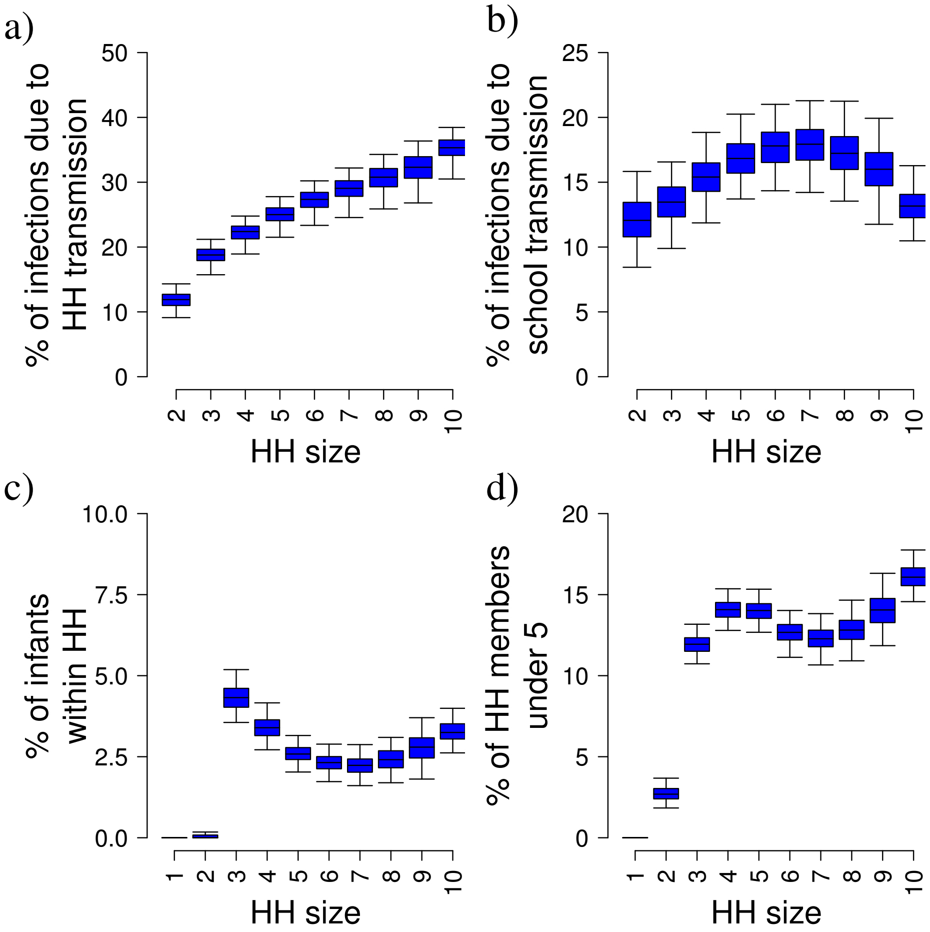

Figure S6: a) Simulated percentage of household members infected within the household for different household sizes. b) Simulated percentage of household members infected through school contacts for different household sizes. c) Percentage of infants in household for different household sizes. d) Percentage of children under 5 year of age in household for different household sizes.

Since in the micro-simulation model each new infection is generated by a specific individual in a specific setting, we recorded exactly the number of infections generated at each time step in each setting, their age and the age of the individual who generated them. Therefore, the percentage of transmission occurring at different settings, the average number of secondary case per setting generated by each infected individual and the proportion of infection episodes occurring due to contacts between two age classes were computed through straightforward computations.

2.1 The role played by household size

The different contribution of household and school contacts in RSV transmission for individuals who belong to households of different size was investigated by tracking for each

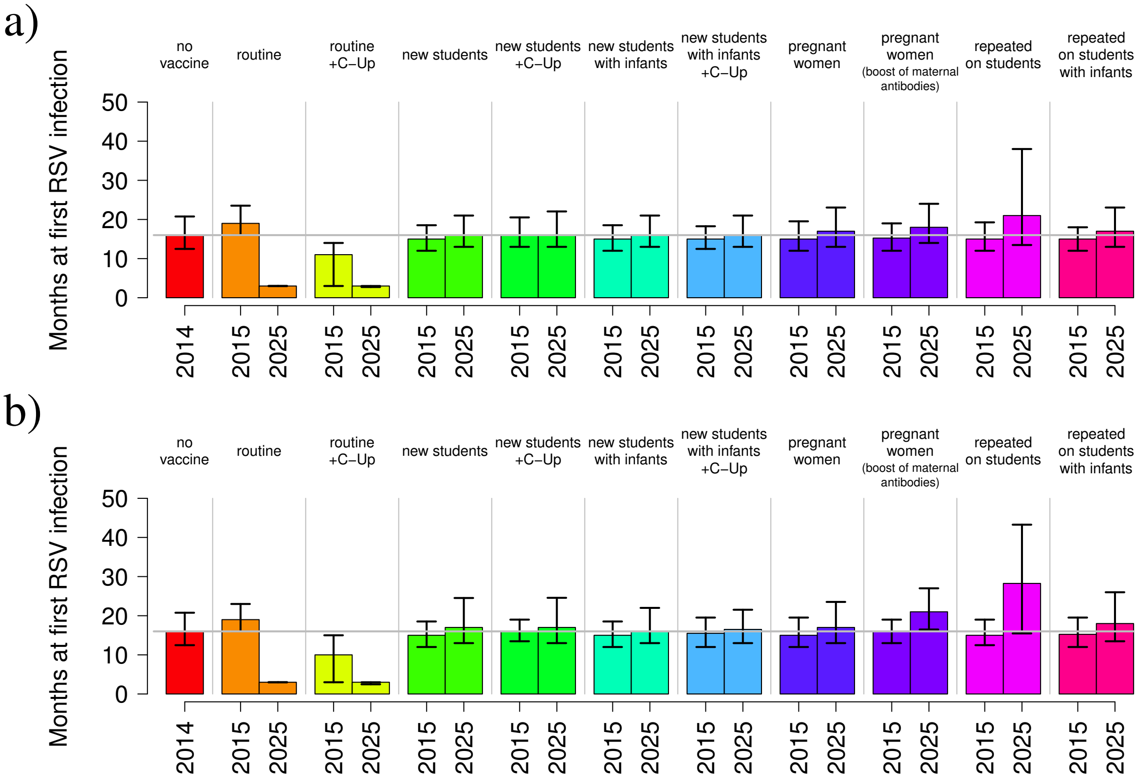

Figure S7: Median age at first RSV infection (in months) among naive individuals after 1 and 10 years of vaccination (2015, 2025), under the assumption of 100% of coverage as obtained by assuming a duration of vaccine protection of 6 months (first row) and, respectively, 8 months for vaccination of pregnant women and 1 year for all the remaining vaccination targets (second row).

household the fraction of cases generated through contacts between household members and schoolmates. Obtained results are shown in Fig.S6a,b. As expected, the fraction of cases generated within households is predicted to increase with household size (see Fig.S6a). Interestingly, the contribution of school transmission is instead larger for individuals belonging to medium size households with respect to small and big houses (see Fig.S6b). This is possibly due to the fact that small households are mainly composed by one or two adults and children before schooling age, while large households are often composed by at least 3 generations with a remarkable fraction of adults and pre-school children (see Fig.S6c,d).

2.2 Age at first RSV infection

The effect of different vaccination strategies on the age at first RSV infection among those who have never experienced neither natural RSV infection nor RSV vaccination after 1 and 10 years of vaccination is shown in Fig.S7 under the assumption of 100% of coverage and two scenarios for the duration of vaccine immunity. Our predictions show that the median age at first infection is lowered by the routine immunization at 3 months of age,

as a consequence of the fact that median age at first infection is computed only among individuals that gets infected before vaccination. However, it is important to stress that the proportion of infants who experience RSV infection before 3 months of age is reduced by 23% thanks to routine immunization. On the opposite side, a remarkable increase of the median age at first infection results as consequence of the yearly vaccination of students and of vaccination of pregnant women when their vaccination is able to increase the duration of maternal antibodies in their newborns. All other vaccination strategies result only in a slight change in the median age at infection with respect to the pre-vaccination period.

3 RSV infection incidence in adults

The predicted incidence by age (including adults) is shown In the main text. Unfortunately, to the best of our knowledge, data on incidence of infection rather than disease are rare and only few attempts have been made to study the infection in older age groups. As a consequence, it is difficult to obtain pristine infection incidence estimates in older children or adults to validate model estimates. However, we performed a supplementary analysis, by comparing the incidence predicted by our model in different age groups with available data on RSV infection in adults. In particular, we attempted to compare model predictions with data from a household study conducted in the epidemic of 2009/2010 in Kilifi, where 44 households, chosen among those with at least one infant and one older sibling <13y, were followed for an entire RSV season for investigating the source of RSV infection in infants. More specifically, we randomly selected 44 households among all simulated households with at least one infant and one older sibling <13y and then estimated incidence rates at different ages and compared them with available data. This procedure was repeated 50 times for each of 1,000 simulated epidemics in order to explore the variability of obtained estimates related to the selected set of households.

The performed analysis suggests that the model is able to produce age-specific infection incidence rates similar to the observed ones when specific households are selected (see Fig.S8). However, the carried out analysis also suggests that an extremely high variability in infection incidence rates by age is expected when estimates are obtained by analyzing a small number of households (<50). Our conclusion is therefore that the data on adults' infection collected in this household study - which was not designed for estimating incidence in adults - are not appropriate neither to calibrate nor to validate our transmission model. This is the reason why we considered only data on children for model parameterization.

4 Sensitivity analysis on transmission rates by settings

In this work we assume that the transmission rate is setting independent, which means that: 1) the individual contact rate is assumed to be the same among different settings; 2) each contact between a susceptible and an infected individual generates a new infection with a probability that does not depend on the setting where the contact occurs.

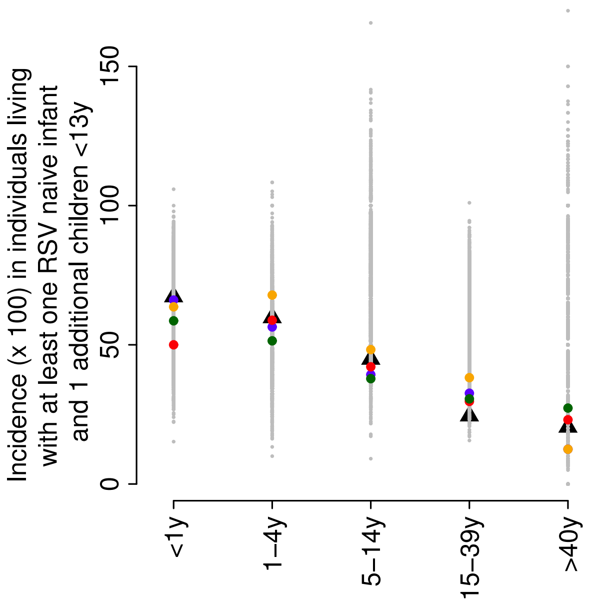

Figure S8: Black triangles represent estimates obtained by infection episodes detected in the household study performed during the epidemic of 2009/2010; different estimates of incidence by age as obtained by different household synthetic studies through model simulation are represented by grey points; illustrative cases have been chosen in order to show that the model estimates are potentially compliant with observed data: points of the same color refer to incidence estimates by age based on the same specific household synthetic study (e.g. red points are associated to estimates of age-specific infection incidence as obtained in the 271st realization of the syntetic household study).

The explicit inclusion of different transmission rates among settings would represent a remarkable model refinement but would also require more detailed epidemiological data to robustly estimate the relative levels of transmission in different settings.

In the main text we assume a RSV transmission rate that is setting independent. To evaluate the robustness of our main findings, we consider different illustrative scenarios in which the relative size of transmission rates in school and in the general community, with respect to the transmission rate in households is a priori assumed and we re-estimated the free epidemiological parameters. In particular the scenarios considered in this analysis have been defined as follows:

- we assume the within-school transmission rate (β_S) to be 1.5 and 2 times the household transmission rate (β_H);
- we assume the transmission rate in the general community (β_G) to be 0.75 and 0.5 times the household transmission rate;
- we assume the transmission rate in the general community to be 0.75 times the household transmission rate and the within-school transmission rate to be 1.5 times the household transmission rate;

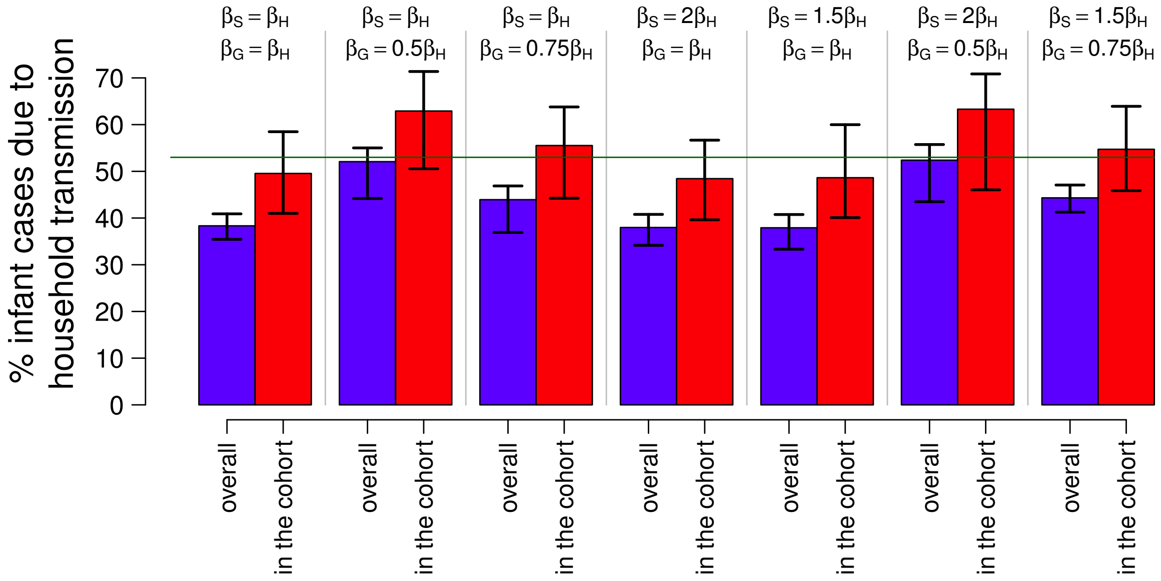

Figure S9: Percentage of infant infections due to household transmission (in all household types (blue) and in the case where the source of infection is defined as in [13] (red)) as obtained under different assumption on the household transmission rate (β_H), the school transmission rate (β_S) and the transmission rate in the general community (β_G). The scenario of a setting independent transmission corresponds to the assumption that $\beta_H = \beta_S = \beta_G$.

- we assume the transmission rate in the general community to be 0.5 times the household transmission rate and the within-school transmission rate at school to be 2 times the household transmission rate.

These results have been compared to the results presented in the main text in terms of 1) the percentage of infant primary infection generated by household transmission and 2) the reduction of the infection incidence among infants and in the general population and the number of vaccine doses administered after 1 and 10 years from vaccination.

Our analysis shows that the estimated contribution of household transmission in generating infant primary infections is stable under different assumption on the transmission rate per settings (Fig.S9). It is also worth of noting that different assumptions on the transmission rate per settings do not significantly affect the effectiveness of immunization strategies considered. Specifically, figure S10 shows the effectiveness of different strategies as obtained by our baseline assumption of a setting independent transmission rate while figures S11, S12, S13, S14, S15, S16 show the effectiveness of different strategies as obtained by simulating the six illustrative scenarios on setting-specific transmission rates described above. Our results show that the set of strategies resulting effective in preventing RSV infant infection remains the same for all the simulated scenarios.

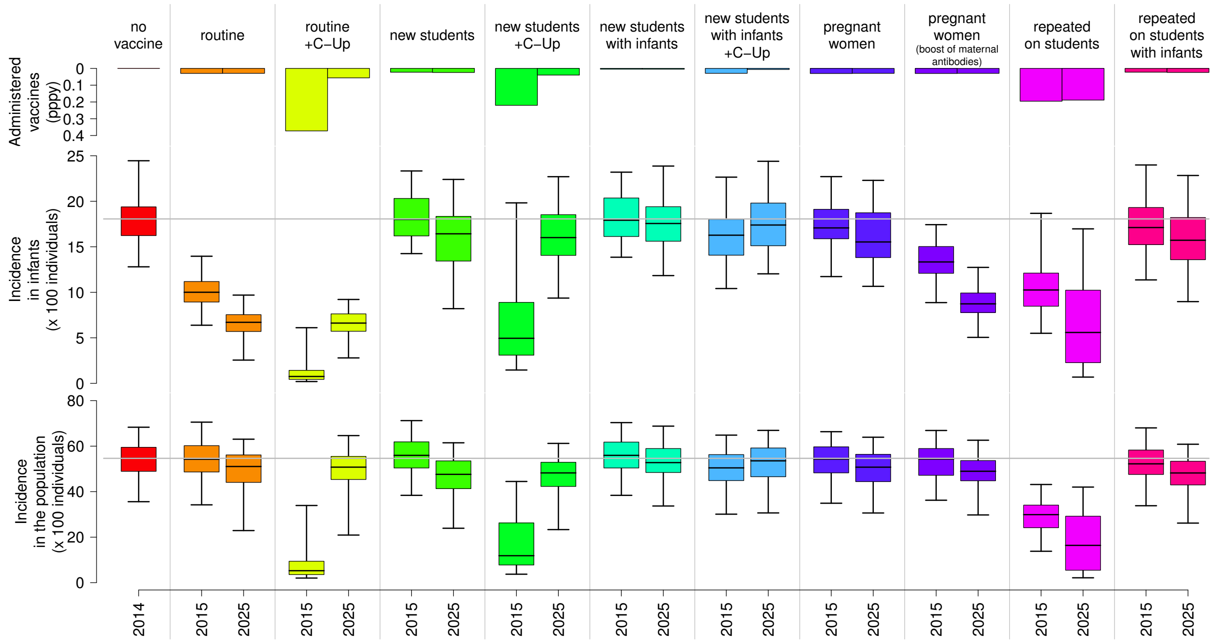

Figure S10: Baseline scenario. Average number of administered vaccine per person per year (top row), boxplot (2.5%, 25%, 75% and 97.5% quantile and mean) of RSV incidence in infants (middle row) and in the general population (bottom row) as predicted by model simulation before vaccination (yr. 2014) and after 1 and 10 years of vaccination (yrs. 2015 and 2025) associated to the considered vaccination strategies under the assumption that $\beta_S = \beta_G = \beta_H$. The gray line reported throughout is used as a reference indicator for the no vaccination scenario.

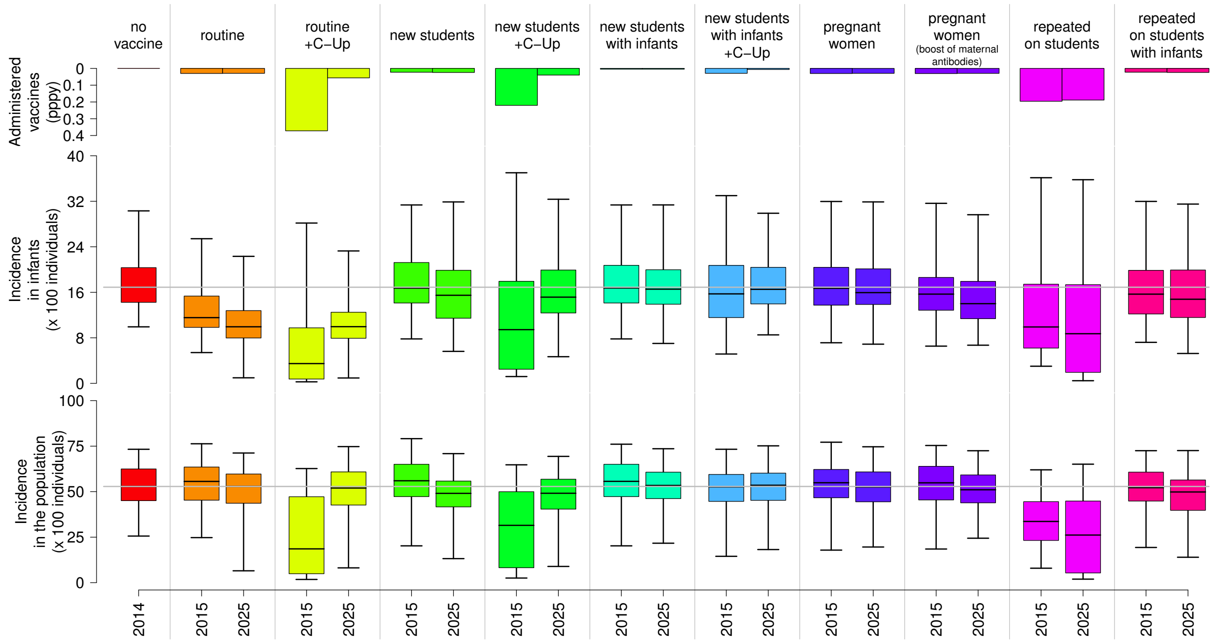

Figure S11: Average number of administered vaccine per person per year (top row), boxplot (2.5%, 25%, 75% and 97.5% quantile and mean) of RSV incidence in infants (middle row) and in the general population (bottom row) as predicted by model simulation before vaccination (yr. 2014) and after 1 and 10 years of vaccination (yrs. 2015 and 2025) associated to the considered vaccination strategies under the assumption that $\beta_S = \beta_H, \beta_G = 0.5\beta_H$. The gray line reported throughout is used as a reference indicator for the no vaccination scenario.

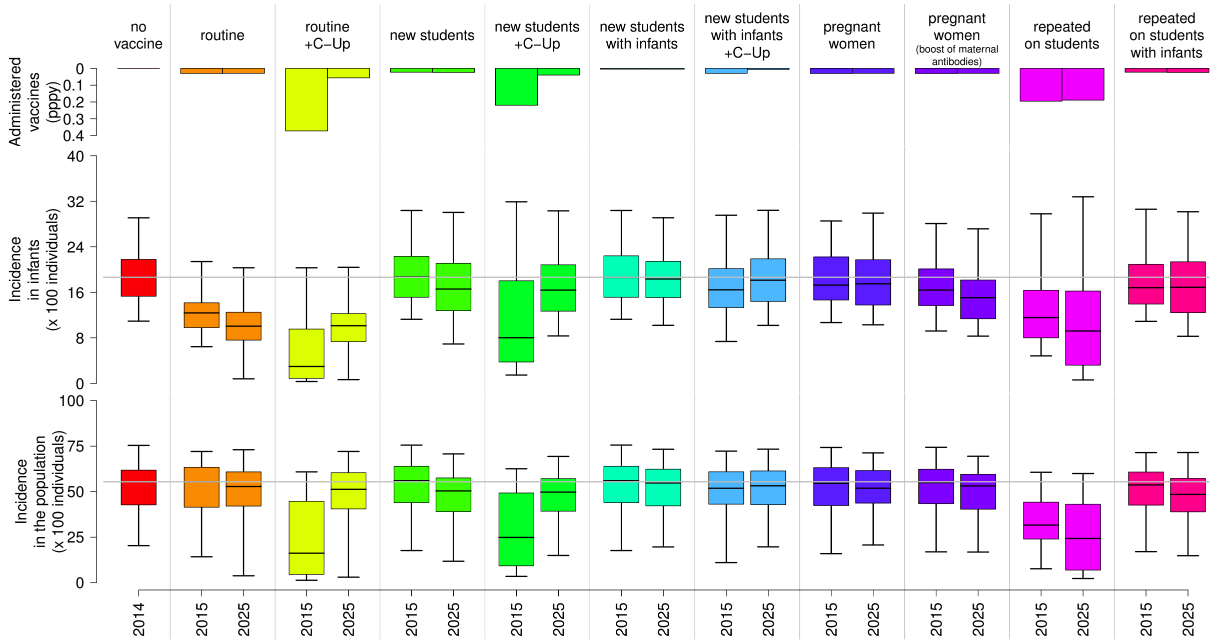

Figure S12: Average number of administered vaccine per person per year (top row), boxplot (2.5%, 25%, 75% and 97.5% quantile and mean) of RSV incidence in infants (middle row) and in the general population (bottom row) as predicted by model simulation before vaccination (yr. 2014) and after 1 and 10 years of vaccination (yrs. 2015 and 2025) associated to the considered vaccination strategies under the assumption that $\beta_S = \beta_H, \beta_G = 0.75\beta_H$. The gray line reported throughout is used as a reference indicator for the no vaccination scenario.

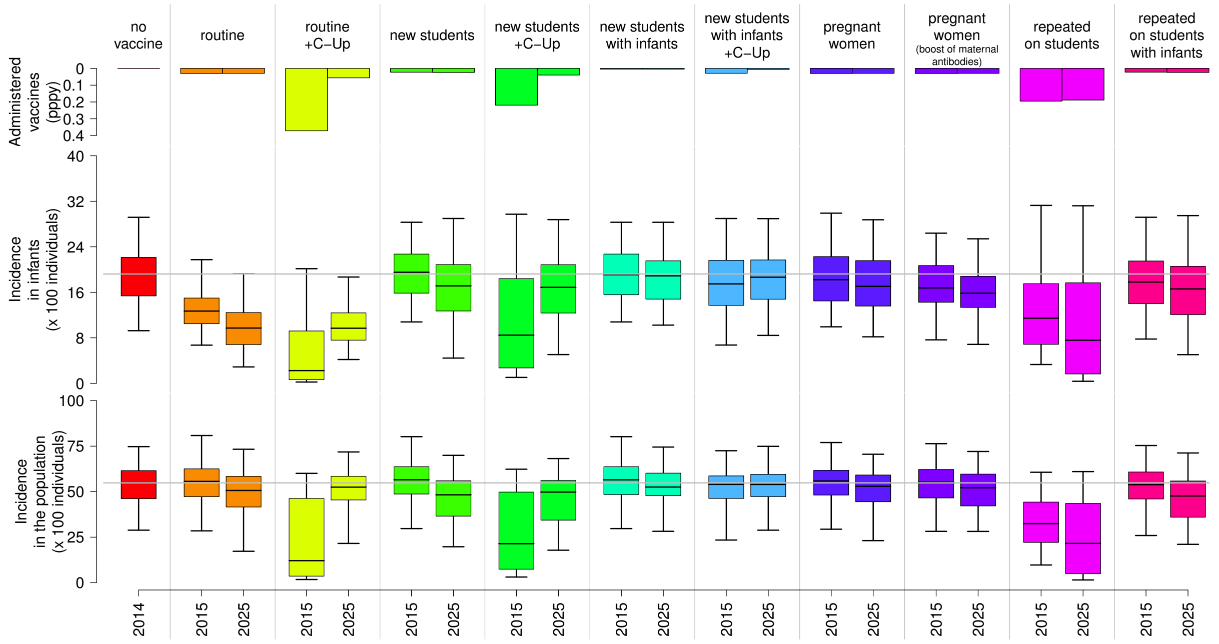

Figure S13: Average number of administered vaccine per person per year (top row), boxplot (2.5%, 25%, 75% and 97.5% quantile and mean) of RSV incidence in infants (middle row) and in the general population (bottom row) as predicted by model simulation before vaccination (yr. 2014) and after 1 and 10 years of vaccination (yrs. 2015 and 2025) associated to the considered vaccination strategies under the assumption that $\beta_G = \beta_H, \beta_S = 1.5\beta_H$. The gray line reported throughout is used as a reference indicator for the no vaccination scenario.

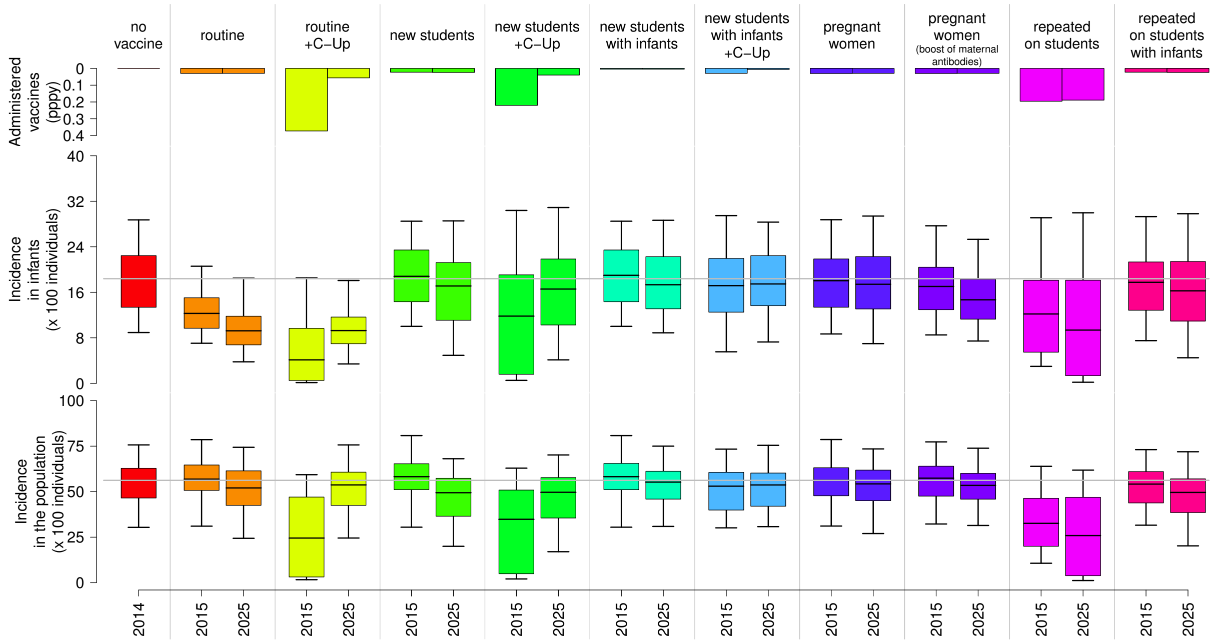

Figure S14: Average number of administered vaccine per person per year (top row), boxplot (2.5%, 25%, 75% and 97.5% quantile and mean) of RSV incidence in infants (middle row) and in the general population (bottom row) as predicted by model simulation before vaccination (yr. 2014) and after 1 and 10 years of vaccination (yrs. 2015 and 2025) associated to the considered vaccination strategies under the assumption that $\beta_G = \beta_H, \beta_S = 2\beta_H$. The gray line reported throughout is used as a reference indicator for the no vaccination scenario.

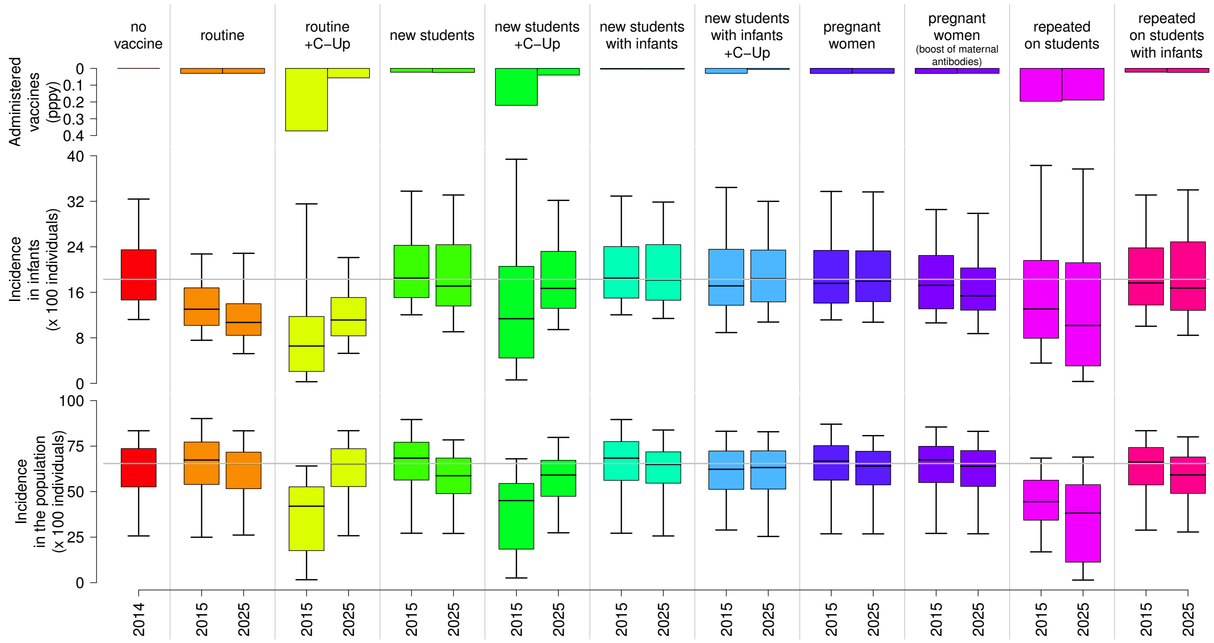

Figure S15: Average number of administered vaccine per person per year (top row), boxplot (2.5%, 25%, 75% and 97.5% quantile and mean) of RSV incidence in infants (middle row) and in the general population (bottom row) as predicted by model simulation before vaccination (yr. 2014) and after 1 and 10 years of vaccination (yrs. 2015 and 2025) associated to the considered vaccination strategies under the assumption that $\beta_G = 0.5\beta_H, \beta_S = 2\beta_H$. The gray line reported throughout is used as a reference indicator for the no vaccination scenario.

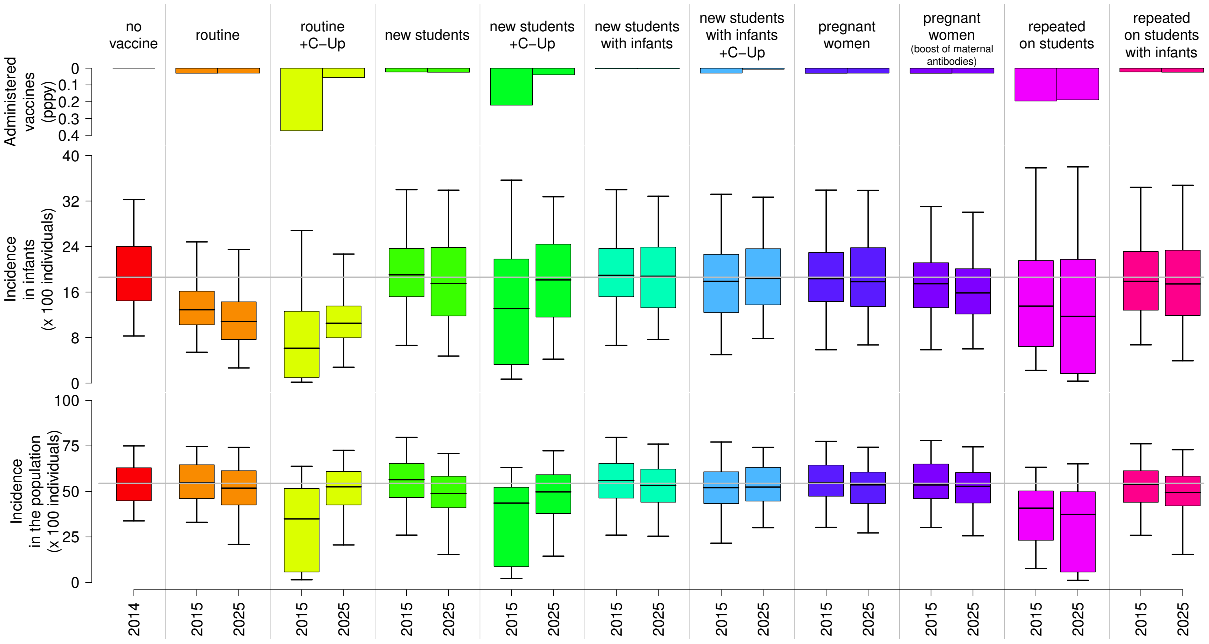

Figure S16: Average number of administered vaccine per person per year (top row), boxplot (2.5%, 25%, 75% and 97.5% quantile and mean) of RSV incidence in infants (middle row) and in the general population (bottom row) as predicted by model simulation before vaccination (yr. 2014) and after 1 and 10 years of vaccination (yrs. 2015 and 2025) associated to the considered vaccination strategies under the assumption that $\beta_G = 0.75\beta_H, \beta_S = 1.5\beta_H$. The gray line reported throughout is used as a reference indicator for the no vaccination scenario.

References

[1] The Demographic and Health Surveys (DHS) Program (2003). Standard DHS. http://dhsprogram.com/data/. Accessed Jun 2014.

[2] Kenya Open Data (2014). Kenya Open Data Survey 2014. http://opendata.go.ke. Accessed Jun 2014.

[3] Ohuma EO, Okiro EA, Ochola R, Sande CJ, Cane PA, et al. (2012) The Natural History of Respiratory Syncytial Virus in a Birth Cohort: The Influence of Age and Previous Infection on Reinfection and Disease. Am J Epidemiol 176: 794–802.

[4] United Nations, Department of Econmic and Social Affaris (2012). World Population Prospects of the United Nations. http://www.un.org/en/development/sea/population/. Accessed Jun 2014.

[5] Gilks WR, Richardson S, Spiegelhalter DJ (1996) Markov Chain Monte Carlo in Practice. Chapman and Hall: London.

[6] Roberts GO, Gelman A, Gilks W (1997) Weak convergence and optimal scaling of random walk Metropolis algorithms. Ann Appl Probab 7: 110–120.

[7] Dorigatti I, Cauchemez S, Pugliese A, Ferguson NM (2012) A new approach to characterising infectious disease transmission dynamics from sentinel surveillance: Application to the Italian 20092010 A/H1N1 influenza pandemic. Epidemics 4: 9–21.

[8] Cauchemez S, Donnelly CA, Reed C, Ghani AC, Fraser Cea (2009) Household transmission of 2009 pandemic influenza A (H1N1) virus in the United States. N Engl J Med 361: 2619–2627.

[9] Cauchemez S, Valleron AJ, Boelle PY, Flahault A, Ferguson NM (2008) Estimating the impact of school closure on influenza transmission from Sentinel data. Nature 452: 750-754.

[10] Merler S, Ajelli M, Pugliese A, Ferguson NM (2011) Determinants of the spatiotemporal dynamics of the 2009 H1N1 pandemic in Europe: implications for real-time modelling. PLOS Comput Biol 7: e1002205.

[11] Chowell G, Hengartner NW, Castillo-Chavez C, Fenimore PW, Hyman JM (2004) The basic reproductive number of Ebola and the effects of public health measures: the cases of Congo and Uganda. J Theor Biol 229: 119–126.

[12] Ferguson NM, Cummings DAT, Fraser C, Cajka JC, Cooley PC, et al. (2006) Strategies for mitigating an influenza pandemic. Nature 442: 448–452.

[13] Munywoki PK, Koech DC, Agoti CN, Lewa C, Cane PA, et al. (2013) The Source Of Respiratory Syncytial Virus Infection In Infants: A Household Cohort Study In Rural Kenya. J Infect Dis doi:10.1093/infdis/jit828.
